# Impacts of two-year multisectoral cash plus programs on young adolescent girls’ education, health and economic outcomes: Adolescent Girls Initiative-Kenya (AGI-K) randomized trial

**DOI:** 10.1186/s12889-021-12224-3

**Published:** 2021-11-24

**Authors:** Karen Austrian, Erica Soler-Hampejsek, Beth Kangwana, Yohannes Dibaba Wado, Benta Abuya, John A. Maluccio

**Affiliations:** 1 Population Council, Avenue 5, 3rd Floor, Rose Avenue, PO Box 17643-00500, Nairobi, Kenya; 2 Independent Consultant, Barcelona, Spain; 3 African Population and Health Research Center, APHRC Campus, 2nd Floor, Manga Close, Off Kirawa Road, P.O. Box 10787-00100, Nairobi, Kenya; 4 Department of Economics, Middlebury College, 14 Old Chapel Road, Middlebury, VT 05753 USA

**Keywords:** Multisectoral, Adolescent girls, Randomized trial, Kenya, Cash transfer

## Abstract

**Background:**

Early adolescence is a critical window for intervention when it is possible to lay a foundation for a safe transition to adulthood, before negative outcomes occur. The Adolescent Girls Initiative–Kenya randomized trial tested the effects of combinations of interventions for young adolescent girls in two sites – the Kibera informal settlement in Nairobi and rural Wajir County in the Northeastern region.

**Methods:**

The interventions included community dialogues on the role and value of girls (violence prevention), a conditional cash transfer (education), weekly group meetings for girls with health and life skills training (health), and training and incentives for financial literacy and savings activities (wealth creation). Participants were randomized to one of four study arms: 1) violence prevention only, 2) violence prevention and education, 3) violence prevention, education and health or 4) violence prevention, education, health and wealth creation. An intent-to-treat (ITT) analysis was conducted using longitudinal data to estimate the impact of each combination of interventions and various sensitivity analyses conducted addressing potential attrition bias and multiple hypothesis testing concerns.

**Results:**

In Kibera, the education conditional cash transfer had small effects on grade attainment but larger impacts on completion of primary school and the transition to secondary school in the most comprehensive arm; the health intervention improved sexual and reproductive health knowledge and condom self-efficacy; and the wealth intervention improved financial literacy and savings behavior. In Wajir, the education conditional cash transfer increased school enrollment and grade attainment, and the wealth intervention improved savings behavior.

**Conclusions:**

The results indicate that when trying to improve a range of outcomes related to adolescent wellbeing for young girls, a multisectoral intervention with components addressing household economic constraints is a promising approach.

**Trial registration:**

Trial Registry: ISRCTN, ISRCTN77455458. Registered 24/12/2015 - Retrospectively registered.

**Supplementary Information:**

The online version contains supplementary material available at 10.1186/s12889-021-12224-3.

## Introduction

Over the past decade there has been increased focus on investments and programming for adolescents, in particular girls [1–4]. Interventions for adolescent girls have potential for triple benefit—in their current lives, in their future lives and in the lives of their children [5]. A focus on young adolescent girls 10–14 years old can be particularly effective since that period is a potentially critical window of opportunity prior to negative outcomes occurring, and thus can lay a foundation for a healthier and more productive future in later adolescence and early adulthood [6–8].

As both children and females, adolescent girls face intersecting vulnerabilities with those living in marginalized settings at even greater risk [2]. For example, among adolescents 15–19 years old, girls account for over two-thirds of new HIV infections worldwide [9]. Moreover, 11% of all births are to girls in that age range, with 95% of those births in low and middle income countries [10]. This is especially concerning since complications during pregnancy and childbirth are leading causes of death for females 15–24 years old [11]. Adolescent girls are also at risk of experiencing emotional, physical and sexual violence, with global estimates indicating that one out of two children 2–17 years old experience some form of violence each year [12]. Although such outcomes are directly related to health, they are also strongly associated with other social factors including inequitable gender norms, unequal access to education—notably secondary school—and early marriage.

Adolescent girls in Kenya face these and similar risks, although the underlying causes and possible remedies may be different across urban and rural areas. Only 57% of girls 15–19 years old living in urban informal settlements are in school [13] and outside urban areas schooling rates are even lower; for example, one-quarter of young adolescent girls in northeastern Kenya have never been to school [14]. In Kenyan urban informal settlements, 9% of girls 15–17 years old and 45% of girls 18–20 have given birth [15]. In northeastern Kenya 50% of girls are married by age 18 [13]. Poor education, health and demographic outcomes, including harmful practices such as female genital cutting and circumcision (FGM/C) and early marriage, are exacerbated by inequitable gender norms and attitudes among adolescents, with over half of very young adolescent girls indicating intimate partner violence is acceptable, similar to rates observed for adult women [13]. Sixteen percent of females in Kenya experience sexual violence before age 18 [16].

Conceptually, in addition to factors at the individual level, factors at the household and community levels also influence adolescent female education, health and economic outcomes. For this reason researchers and practitioners have recommended a socioecological approach for designing effective programs [8, 17, 18]. The socioecological model for adolescent health places individual health, education, self-efficacy and safety at the center, while also incorporating the roles of schools, families and communities. Programs that focus only on the individual may not be able to generate long-term education or fertility benefits, as observed for a recent female empowerment program for vulnerable adolescent girls in Zambia [19].

Relatedly, research also suggests a multisectoral approach—simultaneously addressing education, health and economic constraints—is likely to result in a wider range and longer-lasting set of beneficial outcomes. Substantial literature demonstrates the multiple benefits of educating girls, including improved reproductive health, delayed marriage, lower total fertility and improved health both for the girls and their children, and later economic benefits for her as an adult, as well as for her family and her community [20–23]. Evidence shows economic assets have benefits in girls’ and women’s lives beyond standard poverty measures. Teenage girls in Malawi who received cash transfers for schooling were less likely to begin sexual activity, become pregnant or marry early [24]. Limited economic resources have also been identified as a barrier to safer sex practices and a factor associated with increased transactional sex for adolescent girls [25–28]. Economic interventions on their own, however, may not always improve health outcomes and in some contexts can even increase risk among adolescents [29, 30]. Programs that combine economic strengthening interventions with prevention of violence and health promotion components have had beneficial effects in all three areas [31], although there may be tradeoffs in combining them if they increase costs or if implementation quality is reduced in more complex multisector interventions.

Given the role of poverty in development, cash transfers, both conditional and unconditional, have also become a central component in a wide range of program interventions. It has been established that household cash transfers can have benefits for adolescents, including reducing child labor [32], improving educational outcomes [33–36] and lowering the odds of sexual activity [37] including risky sexual behaviors [38–40] and pregnancy [41]. However, whether cash transfers on their own can have transformative effects is less clear [42, 43], especially because evidence has emerged that cash alone can fall short in promoting longer-term secondary outcomes such as improved learning outcomes or reduced morbidity [44, 45]. This has led to consideration of “cash plus” programming, building on the hypothesis that cash transfers, combined with additional program components or links to external services, can be more effective than cash alone for achieving sustained effects [46]. While such combined social protection has been shown to lead to larger reductions in HIV risk for adolescent girls in South Africa [47], and there is qualitative evidence of increased effectiveness from a program combining cash transfers and financial education for out of school adolescent girls in rural Tanzania [48], research comparing cash alone to cash plus programming for similar populations remains limited.

The Adolescent Girls Initiative-Kenya (AGI-K) is a randomized trial designed to test the short-term (after 2 years) and longer-term effects of two-year, multilevel and multisectoral programs for young adolescent girls 11–14 years old in two different marginalized areas of Kenya. The principal short-term hypotheses analyzed in this paper at the end of the two-year programs in each study site are whether: 1) the different intervention packages affected their targeted outcomes; 2) the more sectors an intervention package covered, the wider the range of outcomes influenced; and 3) compared to a conditional cash transfer (CCT) for schooling, intervention packages that combine a CCT for schooling with health and financial education components, or “cash plus,” result in larger effects on the targeted health and financial-related outcomes specific to the interventions as well as on other outcomes (e.g., schooling) due to complementarities among the interventions. We report results for the two sites in one paper to allow for comparisons of differences in program effectiveness across the two different contexts.

## Methods

### Intervention contexts

**Fig. 1:**
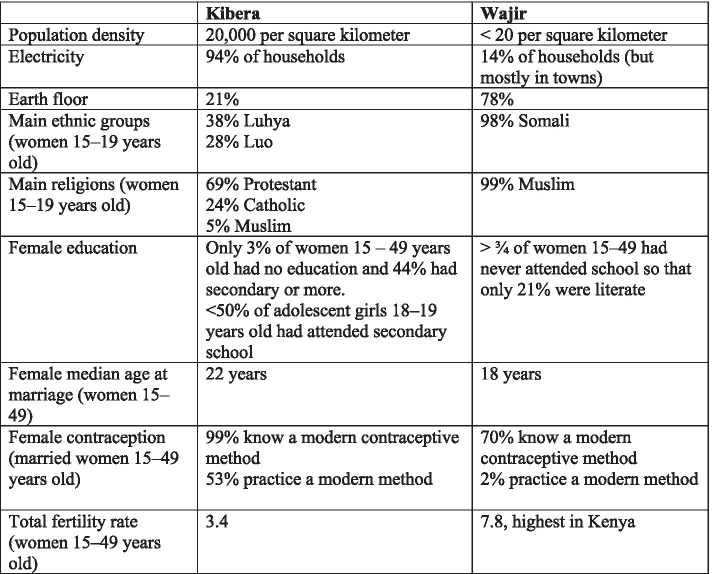
Intervention Contexts

AGI-K was implemented in two different sites: 1) the urban informal settlement of Kibera in Nairobi; and 2) rural Wajir County on the northeastern border with Somalia. Figure 1 summarizes various aspects of the two settings, demonstrating that although quite different both represent marginal populations [13, 15, 49, 50].

Kibera is the largest informal settlement in Kenya and is characterized by high population density with substantial residential mobility, and has poor-quality housing, high crime rates, minimal government services, and multiple ethnic and religious groups. The settlement exhibits characteristics and deprivations similar to other urban settings in Kenya [14] as well as to other urban areas around the continent [51]. In 2006, 60% of adolescent girls 10–19 years old in Kibera felt there was a lot of crime in their neighborhood and feared they would be sexually assaulted [52].

Wajir County is a semi-arid rural area where the principal economic activity is pastoralism (and there is minimal crop-based agriculture) and characterized by low population density, dispersed communities, minimal infrastructure, and a predominant religious and ethnic group, Muslim Somalis [53]. Wajir County is similar in both culture and geography to the other counties of northeastern Kenya, as well as to other semi-arid regions throughout the Horn of Africa.

### Theory of change

**Fig. 2:**
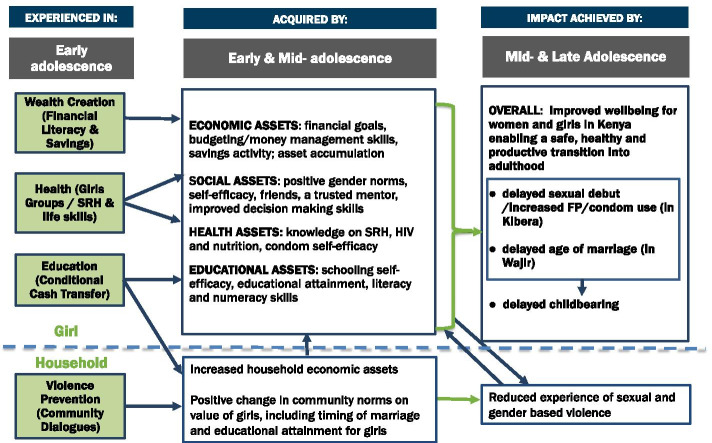
AGI-K Theory of Change

Figure 2 presents the theory of change underpinning AGI-K [54]. It outlines how interventions in the violence prevention and education sectors targeting the community and the household are combined with interventions in the education, health and wealth creation sectors targeting the individual girl. The interventions are designed to work in concert to empower the girls, improving their “ability to formulate strategic choices, and to control resources and decisions that affect important life outcomes” [55]. We hypothesize the interventions affected household norms, household economic assets and adolescent female educational, health, social and economic assets. Violence prevention, resulting from improved resources and norms regarding the value of girls, underpins and complements the effects in other sectors making it beneficial to include it in all study arms.

Important in their own right, these short-term outcomes are also key mediating factors for the primary objective of AGI-K, delayed childbearing. Given the differences across the two sites, effects of the interventions on the mediating factors and the pathways from those factors to delayed childbearing in each setting also likely differ. For example, because initial schooling attainment was substantially different in the two settings, impacts on education, as well as how it subsequently influences childbearing, likely differ. Norms regarding sex and marriage also differed across the settings, with premarital sex common in Kibera but not in Wajir. Therefore, delayed childbearing is hypothesized to result from delayed sexual debut and increased contraceptive use in Kibera, but from delayed marriage in Wajir. This paper assesses the impact on the mediating factors just after the completion of the two-year AGI-K interventions, when girls were 13–16 years old. Arguably, impacts on some or all those factors are necessary for delayed childbearing, the longer-term primary objective to be examined when the girls are older, starting with a subsequent survey 2 years after the end of the interventions (and thus 4 years after the start of the interventions) when the girls would be between 15 and 18 years old.

### Interventions

The AGI-K intervention packages included nested combinations of four single-sector interventions—violence prevention, education, health and wealth creation—implemented for 2 years from August 2015 through July 2017. The Population Council Kenya oversaw the programs which were implemented and comprehensively monitored by the non-governmental organizations (NGOs) Plan International in Kibera and Save the Children in Wajir. Below we describe the interventions in detail and use administrative data collected during program monitoring by the implementing NGOs to summarize key measures of implementation and take-up for each intervention, demonstrating that fidelity of the interventions was high.

**Fig. 3:**
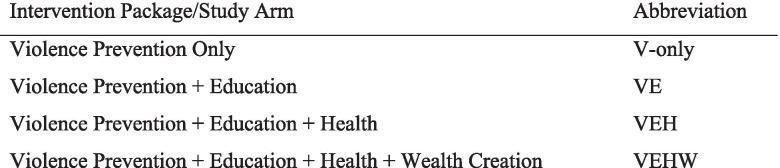
Intervention Packages/Study Arms

**Fig. 4:**
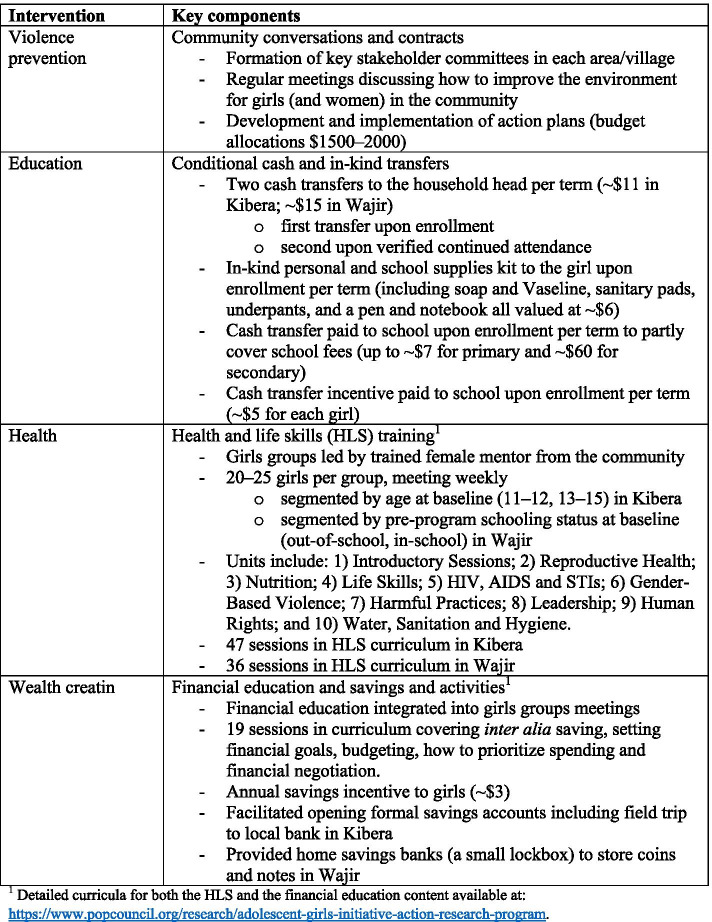
AGI-K Intervention Components

Rather than examine each single-sector intervention in isolation, because the theory of change posits complementarities between the different sectors, the study examined the effectiveness of different multisectoral packages of interventions, compared with a base intervention addressing violence. Girls were randomized (described below) to one of the four combinations of interventions, or study arms. All four packages included the community-level violence prevention intervention so that there is no pure control group (we return to the implications of this aspect of the study design below). In Kibera this was necessary because the violence prevention intervention operates at a geographic level and it was not feasible to identify a large number of distinct geographic locales to yield a study with adequate power. In Wajir, a condition of local authorities to carry out the study was that all participating villages benefit. The mix of intervention packages was the same in Kibera and Wajir, although several aspects of the actual interventions implemented were modified to suit the different sites. Figure 3 summarizes the study arms and Fig. 4 outlines the key elements of the interventions.

The violence prevention intervention employed community conversations and contracts [56]. The conceptualization of violence was not limited to sexual and physical violence but incorporated the undervaluation of girls reflected by lack of education, FGM/C and early marriage. Implementing NGOs first established a committee of key stakeholders in each community and facilitated a series of dialogues to identify local problems and constraints contributing to the undervaluation of girls and to violence against girls and women. The committees then developed action plans and a proposed budget to address causes and alleviate some of the local challenges facing adolescent girls. The intervention was designed to reduce violence against women and girls by improving local norms and augmenting resources available to address local challenges.

In Kibera, committees began meeting in December 2015 and met on average 30.0 (SD 9.4) times through July 2017, by which time they had completed their action plans. Plans focused on setting up new resource centers or libraries for girls in the community. In Wajir, committees met on average 15.6 (SD 10.4) times over the intervention period, although in a few communities there were less than five organized meetings (and on average there were four fewer meetings in V-only clusters). By November 2017, 68 of 79 action plans had been completed. Plans mostly focused on improving village primary schools, for example purchasing additional desks, installing piped water or solar panels, or building new classrooms.

The education intervention included cash and in-kind transfers conditional on enrollment of the target girl in each of the three school terms per year and on her regular attendance during the terms. Although the school year in Kenya begins in January, enrollment is permitted at the start of each term. All girls were eligible for transfers upon confirmation of enrollment, regardless of whether they were in school prior to the program.

At the start of each school term, the implementing partners verified enrollment and based on that the program delivered all but the second installment of the household-level transfer which was conditional on attendance during the term. Biometric fingerprint reading devices were initially used to monitor attendance at each school (over 200 in Kibera and 79 in Wajir). Technical and logistical problems (including difficulties with electronic data transfer, substantial delays as students waited to comply and lack of cooperation by some schools) led to incomplete implementation of the biometric system by school staffs. Consequently, the attendance conditionality was not enforced during the first two terms to ensure transfers were not withheld due to missing biometric information for girls who in fact had been attending. After two terms the biometric system was discontinued, a deviation from the original program design. From then on attendance was monitored (and conditionality applied) each term during two random visits to the school, 1 week apart. Girls absent from school during both visits were ineligible for the second household-level cash transfer payment for that term.

Take-up of the education intervention was high in both Kibera and Wajir (Table 1). Ninety percent or more of girls randomized to a study arm including the education intervention received at least one household cash transfer. Out of a possible 12 transfers, girls received on average 9.5 (SD 3.7) in Kibera and 8.8 (SD 4.3) in Wajir (Table 1). By design, there were no transfers to girls in the V-only study arm.

**Table 1:** AGI-K Intervention uptake, by site and study arm

	Kibera					Wajir				
	V-Only	VE	VEH	VEHW	Overall^a^	V-Only	VE	VEH	VEHW	Overall^a^
Education intervention										
Received at least one cash transfer, %	0.0	92.7	90.1	94.6	92.5	0.0	88.2	87.5	89.9	88.5
Cash transfers received (out of 12), mean	0.0	9.4	9.2	9.9	9.5	0.0	8.7	8.7	9.0	8.8
School fee payments received (out of 6), mean	0.0	4.9	4.9	5.0	5.0	0.0	3.9	3.9	3.9	3.9
School kits received (out of 6), mean	0.0	4.1	4.1	4.4	4.2	0.0	4.4	4.3	4.5	4.4
Health intervention										
Total group meetings attended,^b^ mean	0.0	0.0	34.5	37.6	36.0	0.0	0.0	30.8	35.2	33.0
Attended at least 12 group meetings, %	0.0	0.2	77.5	82.8	80.1	0.0	0.0	70.8	72.6	71.7
Health and life skills group meetings, mean	0.0	0.0	34.3	27.0	30.7	0.0	0.0	30.6	21.4	26.0
Wealth creation intervention										
Financial education group meetings, mean	0.0	0.0	0.1	10.5	10.5	0.0	0.0	0.2	13.8	13.8
At least 4 financial education group meetings, %	0.0	0.0	2.1	80.7	80.7	0.0	0.0	0.0	76.2	76.2
Received at least one annual savings incentive, %	0.0	0.0	0.0	81.6	81.6	0.0	0.0	0.0	83.6	83.6
Opened savings account/received home savings bank,^c^ %	0.0	0.5	0.3	81.9	81.9	0.0	0.0	0.0	78.5	78.5
N	597	592	609	592	2390	506	549	538	554	2147

Source is program administrative data collected during program monitoring by the implementing NGOs

^a^ Overall average across applicable study arms (VEH and VEHW for health intervention and VEHW for wealth creation intervention)

^b^ Groups met weekly over 2 years for a maximum of ~ 100 meetings

^c^ Savings accounts in Kibera and home savings banks in Wajir

The health intervention consisted of weekly educational meetings in which designated groups of 20–25 girls met under the guidance of trained female mentors from the community. The mentors were young women 18–30 years old recruited from the same communities as the girls. In Kibera, mentors had completed secondary school and had some prior experience with training adolescents or youth –typically on SRH topics. In Wajir, due to generally lower educational attainment, the education requirement for mentors was primary school completion. The group meetings included facilitated discussions following a comprehensive health and life skills (HLS) curriculum developed by the program with time for open discussion, together aimed at empowering the girls. The HLS curriculum included the same ten units in both sites, but specific content within them differed across the sites (for example with greater emphasis on sexually transmitted diseases and their transmission in Kibera) such that there were 47 unique sessions in the curriculum in Kibera and 36 in Wajir. Once a girls group had completed all of the sessions the mentor could use subsequent meetings to repeat specific sessions.

In Wajir, it was an even more significant challenge than expected for the NGO to identify enough qualified women to serve as mentors, in part because of the low education levels of adult women residing in the communities. Consequently, at the outset there was substantial variation in how well mentors carried out their roles, and not all delivered the curriculum content effectively. To address this, for Wajir only the intervention design was modified and the sessions transformed into recorded audio scripts starting in June 2016. The groups listened together to the recordings, which maintained the discussion format including instructions on when to pause and questions to discuss. Both mentor and participant feedback indicated the modified approach was more successful [57].

Over the course of the intervention, girls randomized to a study arm including the health intervention attended 36.0 (SD 24.0) group meetings on average in Kibera and 33.0 (SD 25.2) in Wajir (Table 1). In both Kibera and Wajir, overall average attendance at the group meetings was modestly higher for girls in the VEHW than in the VEH study arm. Because the total number of scheduled meetings across study arms was the same an implication is that the girls in VEH had greater exposure to the HLS curriculum. By design, girls in the VE (or V-only) study arms were not included in the group meetings though program administrative data indicate that in Kibera one girl in VE did attend.

The final intervention, wealth creation, integrated a 19-session financial education (FE) curriculum into the mentor-led group meetings to promote economic empowerment. Although the total number of sessions was the same, as with HLS, the specific curriculum content differed modestly across Kibera and Wajir. For example, whereas home savings banks were discussed in both sites, formal bank accounts were covered in detail only in Kibera since they were largely inaccessible to girls in Wajir.

Over the course of the intervention, girls randomized to the VEHW study arm attended on average 10.5 (SD 7.0) meetings related to FE in Kibera and 13.8 (SD 9.8) in Wajir (Table 1). By design, the VEH study arm did not include the FE curriculum. Program administrative data, however, indicate that in four villages in VEH a few FE sessions were offered, exposing ~ 10% of girls in that study arm to one of the 19 sessions on FE in error. Approximately 80% of eligible girls received at least one of two annual savings incentive transfers and opened a savings bank in Kibera or had a home bank savings in Wajir.

### Randomization and data collection

The unit of randomization for the packages of interventions (study arms) differed across the two sites with individual-level randomization done in Kibera and cluster-level randomization in Wajir. In densely populated Kibera, with widespread availability of schools in and near the settlement, it was possible to target different girls with the different intervention packages including education, health and wealth creation (although not for the geographically focused violence prevention intervention) so that an individual-level randomized design was feasible. At the same time, the high mobility within Kibera (linked to often uncertain land/house tenure) and school attendance patterns in which girls attend both public and private schools of very different sizes throughout the area and in adjacent areas made it less advantageous to consider a cluster design in this setting [58]. In less densely populated Wajir, with its small, cohesive and geographically separated village settlements, individual-level randomization was not feasible but a cluster-level design allowed for randomization of different interventions at the village level, providing a sample of girls of sufficient size for the study. In Wajir, clusters were defined as village settlements with one public primary school. This ensured that girls had access to a school and to a central location for group meetings (often but not always held in schools). A total of 80 clusters were identified in three (of four) districts as defined in the 2009 national census: 20 clusters in Wajir West, 28 in Wajir East and 32 in Wajir South. For security reasons the program was not implemented in one cluster (VE study arm in Wajir West) and girls in that village were not followed after baseline.

Assignment to study arms was done publicly to ensure transparency and strengthen program acceptance. In Kibera, girls were randomly assigned to study arms in February 2015 during a public meeting attended by local leaders and other stakeholders. A spreadsheet with a line for each eligible girl (identified only by an anonymous ID number) was projected onto a screen and a random number generated for each. The list was then put in ascending order based on the random number and divided into four equally sized groups. Each group was assigned to a study arm when four stakeholders in turn each blindly drew a card from a set of four cards, each indicating one of the study arms. In each of the three districts in Wajir, a public meeting was held April–June 2015 with local leaders and other stakeholders. A list of names of all the clusters in the district was displayed and a representative from each cluster blindly drew a card indicating a study arm from a set with one card available for each cluster in the district, equally divided across the four study arms.

By design, all community members living in the study areas were exposed to the violence prevention intervention comprising facilitated community conversations and subsequent action plans. For the other intervention components, the primary beneficiaries were resident girls 11–14 years old at the start. In Kibera, a complete household listing of the area done prior to randomization and the baseline survey to identified all girls in the age range. Girls were eligible at baseline if they were residing in the study area and not currently enrolled in boarding school, since participation in locally-based meetings would be infeasible for those studying away. One eligible girl per household was selected for the baseline survey (and other eligible girls invited to participate in the program in the same study arm). In Wajir, where the vastness of the terrain complicated survey logistics and increased survey costs, a rapid household listing was conducted in each cluster just prior to the baseline survey. All girls 11–14 years old residing in the village were eligible for the program except for ~ 3% who were currently enrolled in boarding school. In clusters with fewer than 40 households with an eligible girl all households and girls 11–14 years old were selected for the baseline sample interview. In smaller clusters, multiple eligible girls in the same household could be interviewed. In clusters with 40 or more households with an eligible girl, team leaders used predetermined cluster-specific random number lists to randomly select 40 households and one girl within the household for the baseline sample interview. In practice, difficulties confirming accurate ages meant some girls slightly younger than 11 or older than 14 were also included. All eligible girls in the cluster were invited to participate in the program in the same study arm.

The baseline survey was conducted in both sites prior to the start of the intervention, and a follow-up longitudinal survey 2 years later at the end of the interventions. All girls interviewed at baseline were targeted for the two-year follow-up. In Kibera the initial household listing took place November 2014–January 2015, baseline data was collected February–April 2015 and the two-year follow-up survey May–July 2017. In Wajir, baseline data was collected March–May 2015 and the two-year follow-up survey July–September 2017. In Kibera, randomized beneficiaries were not notified until after the baseline survey. In Wajir, randomization to study arms occurred after the baseline survey in each district.

### Outcomes

In this paper we analyze the mediating factors the theory of change predicts would have been impacted during early and mid-adolescence. These represent the *secondary* outcomes of the AGI-K randomized trial outlined in the study protocol [54] and summarized in Appendix Table 1. For some domains we considered additional related variables and in part for this reason we also construct summary measure variables for each domain.

**Fig. 5:**
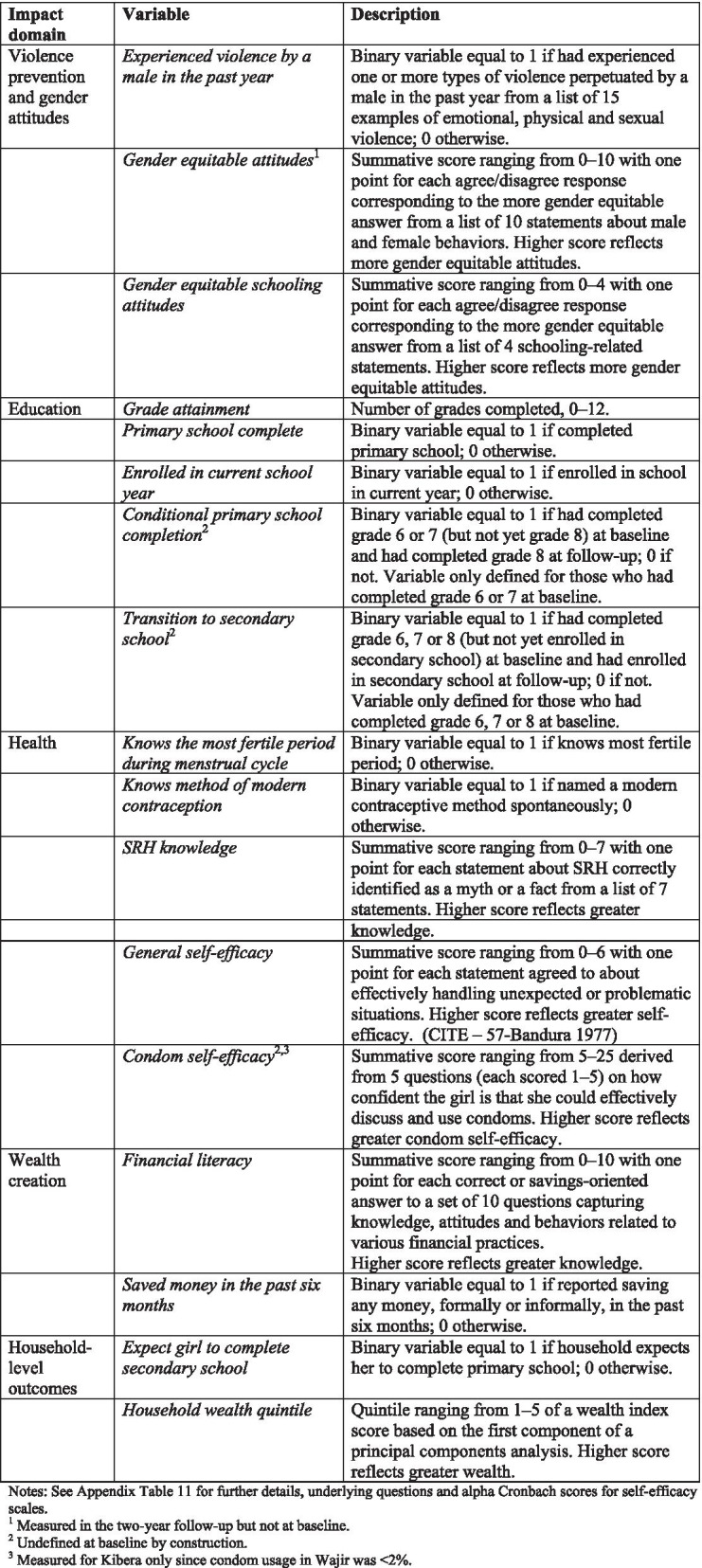
Outcome measures by domain

Our theory of change posits that the single-sector interventions each have potential to influence outcomes across all the domains, particularly if there are synergies or complementarities between intervention components; therefore we measured all outcome variables in all study arms and present estimates for each study arm relative to V-only. The research design does not permit identification of impacts attributable to the violence prevention intervention alone. It is possible, however, to examine whether the other packages of interventions affected violence-related outcomes and gender attitudes above and beyond any effects the V-only intervention may have had. Figure 5 summarizes the outcome variables by domain and Appendix Table 11 provides the full details including the underlying questions and Cronbach alphas for self-efficacy scores.

The theory of change underscores the potential importance of community norms as well as household-level economic resources for schooling, for example to cover school fees and pay other school-related expenses. Although not pre-specified in Appendix Table 1, we used household level measures to assess program impacts on related outcomes including household norms reflected by whether the household head expects the girl to complete secondary school. Last, we examined the first component from a principal components analysis (PCA) estimated using ten variables measuring assets, housing characteristics and cash liquidity to derive a household wealth index [58]. The index was divided into quintiles with higher quintiles representing higher wealth.

### Sample size calculations

The study was powered to detect differences in the number of grades attained and prevalence of first birth between V-only and each of the three other study arms at endline—4 years after the start of the programs when girls in the sample would be 15–18 years old [54]. For Kibera, we estimated endline average grades attained of 8.3 years (and correlation coefficient 0.33) using the 2008–09 Kenyan Demographic and Health Survey (KDHS) [59] and an endline birthrate of 15.4% using the 2012 Nairobi Cross-Sectional Slum Survey (NCSSS) [15] and used a power of 80% to calculate sample size. Based on individual randomization, the estimated final sample size was 600 girls per arm (750 girls per arm at baseline, assuming a loss to follow-up of 20%). Because of a higher-than-expected proportion of ineligible girls after complete enumeration of Kibera, however, the attained baseline sample included approximately 600 girls per arm. After attrition this allows a minimum detectable difference of 0.55 grades of schooling between any two study arms. For Wajir, we estimated endline average grades attained of 2.8 years (and correlation coefficient of 0.26) and an endline birthrate of 17.6% using data from the Northeastern Province from the 2008–09 KDHS [59] and a power of 80% to calculate sample size. Based on cluster randomization, the estimated final sample size was 20 clusters per arm with 32 girls per cluster (40 girls per cluster at baseline, assuming a loss to follow-up of 20%). Because of differences between population estimates and the actual number of eligible girls residing in these communities, however, the attained baseline sample included 20 clusters per arm with an average of 27 girls per cluster [54]. After attrition this allows a minimum detectable difference of 0.49 grades of schooling between any two study arms. Because the estimated models control for additional variables in both trials, the minimum detectable differences may be smaller.

### Statistical methods

Following the analysis plan [54], we estimated the intent-to-treat (ITT) impact of each nested combination of single-sector interventions relative to the V-only arm using ordinary least squares (OLS). For binary outcomes these are linear probability models. ITT was defined as girls randomized to a specific study arm in Kibera, and as girls living at baseline in a village randomized to a specific study arm in Wajir, irrespective of their actual participation in the program. Analysis of covariance (ANCOVA) models controlling for baseline values of the outcome variable when available were used to obtain ITT estimates from the two-year follow-up survey for each site. All scales and summative scores were converted into z-scores, dividing by the standard deviation of the V-only group at baseline when available; otherwise they are divided by the standard deviation of the V-only group at the two-year follow-up.

Because several variables were evaluated, we accounted for multiple hypotheses testing by: 1) grouping outcomes into families by domain and examining summary measures for each domain; and separately 2) recalculating statistical significance. First, for each variable we constructed a z-score based on the mean and standard deviation of the V-only study arm at the two-year follow-up. Using those, we constructed an inverse covariance weighted index for each family of outcome variables, re-standardizing the index to be mean 0 and SD 1 [60]. We then estimated the same ITT model on this summary z-score for each domain. (As an alternative index we calculated the simple average z-score for the set of variables in each domain [61]; results using this alternative summary z-score are substantively the same.) Second, we recalculated statistical significance using the Benjamini and Hochberg (1995) false discovery rates (FDR) and report the adjusted q-values in the appendix, discussing any differences in the text [62]. For each study site, these multiple hypothesis testing corrections are done for all individual items together and, separately, for the four summary measures together.

Prior to presenting the impact estimates, we assessed balance on baseline characteristics across the experimental arms for the non-attriting sample after 2 years to explore potential bias from non-random attrition. In addition to this consideration of baseline balance, we examined the extent and correlates of attrition in the study using OLS to estimate the probability of successful interview in the two-year follow-up for each site and also examined whether the correlates of attrition differed by study arm. We then used OLS stepwise backward elimination with adjusted R^2^ as the information criterion [63] to develop a parsimonious model of attrition and constructed inverse probability weights, reporting weighted regressions in the appendix.

All regressions controlled for age (using a binary variable for each age in years at baseline) and when available the baseline value of the outcome measure. Regressions for Wajir also controlled for stratification with binary variables for the districts. In the appendix, we report adjusted regressions with additional baseline controls for cognitive skills, schooling, parental characteristics and household wealth to account for any initial imbalance and improve precision [54]. Last, in the appendix we also present results combining all three arms with the education intervention (VE, VEH and VEHW) and estimate their average overall treatment compared to V-only. Because the outcomes include both continuous and binary variables, our main specifications use OLS for comparability of reporting estimated coefficients and confidence intervals across the distinct variables as well as the summary z-score measures, all of which are continuous since each domain has at least one continuous variable in it. To examine whether the findings for binary outcomes are sensitive to using a linear probability model, we also estimate logistic models reporting odds ratios and their confidence intervals.

Regressions for the individual-level randomization in Kibera were estimated with robust standard errors and for the cluster-level randomization in Wajir with robust standard errors accounting for clustering at the village level. We define statistical significance at 0.05. Statistical analysis was conducted using Stata 15.1.

## Results

**Fig. 6:**
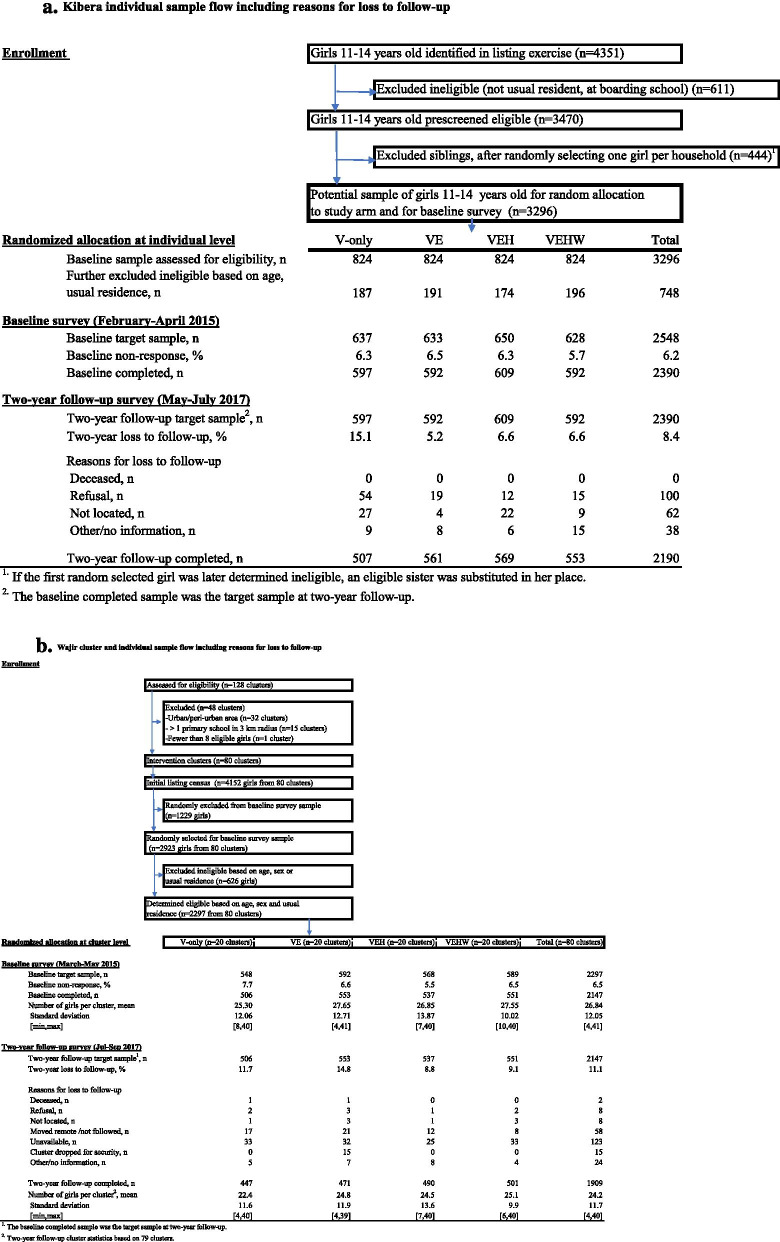
**a** Kibera Sample Flow. **b** Wajir Sample Flow

Figure 6a and b show the sample flow and analytical samples by site and study arm. Updates during fieldwork (particularly in Kibera where there was a lag between the initial listing and the baseline survey) led to reduced samples excluding ineligible girls. Notably, accurately capturing age in both contexts benefited from multiple visits.

The final baseline data included 2390 girls interviewed in Kibera and 2147 girls in Wajir. Baseline survey non-response was 6.2% in Kibera and 6.5% in Wajir and most commonly due to unavailability of the girl for interview (because she was not located, temporarily away or after the rapid listing it was determined that she was not a usual resident) and in some cases (about one-third in Kibera and 10% in Wajir), refusal. Because there was substantial residential mobility for girls in the study sites (particularly in Kibera), to keep attrition at a minimum the two-year follow-up survey tracked girls to their new locations. In Kibera this included following girls to 27 (out of 47) different counties throughout Kenya; about one-fifth of girls re-interviewed in the two-year follow-up were located outside of Nairobi City County. In Wajir mobility was lower and girls were tracked to new locations throughout the county as well as a small number outside the county; approximately 14% were interviewed in a location different from the baseline. At the two-year follow-up in 2017, 91.6% of girls from the Kibera baseline sample were re-interviewed and 88.9% from the Wajir sample. The final analysis sample included 2190 girls from Kibera and 1909 from Wajir. Reasons for loss to follow-up are shown in Fig. 6a and b. The most common were failure to locate girls who had moved or unavailability of the girl during the interview period. Direct refusal was more common in Kibera (particularly in the V-only arm), but rare in Wajir. In Wajir, the 15 girls interviewed at baseline in the VE study arm cluster dropped from the trial due to security concerns were not followed.

Figure 6a and b also show that two-year follow-up rates differed by study arm (85–95%); therefore, we tested baseline balance for selected background characteristics using the non-attritted sample. Table 2 (Kibera) and Table 3 (Wajir) present baseline means for all available outcomes for the sample of girls interviewed at the two-year follow-up (outcomes not included were the new measurements introduced in the two-year follow-up). The samples in the two-year follow-up in each study arm were balanced on a range of baseline characteristics and there were no large differences and only one significant difference for general self-efficacy in Wajir. Appendix Tables 2 and 3 report means by intervention for the full baseline sample [64].

**Table 2:** Kibera baseline means of key variables for analytical sample at two-year follow-up, by study arm

	(1)	(2)	(3)	(4)	(5)
	V-Only	VE	VEH	VEHW	*p*-value
Background					
Age, mean (sd)	12.6 (1.2)	12.5 (1.3)	12.6 (1.3)	12.5 (1.3)	0.707
Cognitive score (0–16), mean (sd) [*n* = 2173]	8.2 (3.0)	8.4 (3.0)	8.3 (3.1)	8.3 (3.2)	0.732
Lives with both parents, % [*n* = 2174]	52.1	55.9	51.0	53.3	0.384
Mother completed primary school, % [*n* = 2040]	62.4	63.3	62.5	64.1	0.938
Father completed primary school, % [*n* = 1803]	77.7	78.9	74.7	79.6	0.305
Violence Prevention					
Experienced violence by a male in the past year, %	29.0	29.8	30.6	32.2	0.703
Positive gender attitudes score (0–4), mean (sd)	3.6 (0.7)	3.5 (0.7)	3.6 (0.7)	3.6 (0.7)	0.458
Education					
Grade attainment, mean (sd)	5.7 (1.4)	5.7 (1.3)	5.7 (1.4)	5.7 (1.3)	0.990
Primary school complete, %	7.9	5.9	6.7	6.1	0.600
Enrolled in school, %	99.2	99.1	98.6	99.3	0.713
Literate in Swahili and English, % [*n* = 2173]	92.2	93.6	92.9	94.0	0.700
Health					
Knows most fertile period during menstrual cycle, %	8.7	8.2	7.0†	5.8	0.243
General self-efficacy score (0–6), mean (sd)	3.8 (1.7)	4.0 (1.6)	3.9 (1.6)	4.0 (1.6)	0.493
Wealth Creation					
Financial literacy score (0–10), mean (sd)	5.8 (1.9)	5.6 (1.9)	5.7 (1.9)	5.8 (1.9)	0.426
Saved money in the past 6 months, %	27.8	25.7	26.4	28.4	0.722
Household-level					
Household expects girl to complete secondary, % [*n* = 2222]	99.8	99.6	99.8	99.8	0.934
Household wealth quintile (1–5), mean (sd) [*n* = 2236]	3.1 (1.4)	3.1 (1.4)	3.0 (1.4)	3.0 (1.4)	0.631
Sample by arm when *n* = 2190	507	561	569	553	2190

Asterisks in columns 2–4 indicate statistically different from V-only. *P*-values in column 5 are from an F test for joint differences across study arms for sample of *N* = 2190 (unless otherwise noted) non-attritors in the two-year follow-up. All statistical tests carried out using robust standard errors

*** *p* < 0.001, ** *p* < 0.01, * *p* < 0.05, † *p* < 0.1

**Table 3:** Wajir baseline means of key variables for analytical sample at two-year follow-up, by study arm

	(1)	(2)	(3)	(4)	(5)
	V-Only	VE	VEH	VEHW	*p*-value
Background					
Age, mean (sd)	11.9 (1.3)	12.0 (1.3)	11.8 (1.2)	11.8 (1.3)	0.128
Cognitive score (0–16), mean (sd) [*n* = 1874]	5.3 (2.9)	5.0 (3.0)	5.0 (3.2)	5.4 (3.0)	0.577
Lives with both parents, % [*n* = 1892]	73.6	73.9	76.2	73.7	0.846
Mother ever attended school, % [*n* = 1892]	1.4	1.5	0.6	0.4†	0.193
Father ever attended school, % [*n* = 1888]	5.7	2.8†	3.3	3.2	0.314
Violence Prevention					
Experienced violence by a male in the past year, % [*n* = 1883]	4.1	3.9	3.1	2.2	0.444
Positive gender attitudes score (0–4), mean (sd) [1883]	3.0 (1.0)	2.8 (1.1)	2.9 (1.1)	2.9 (1.1)	0.509
Education					
Grade attainment, mean (sd)	3.1 (2.1)	2.8 (2.3)	2.8 (2.2)	2.6 (2.1)	0.467
Primary school complete, %	1.3	0.6	0.8	0.8	0.890
Enrolled in school, %	83.4	72.8*	79.6	74.1†	0.141
Literate in Swahili and English, % [*n* = 1874]	41.6	34.8	40.3	34.5	0.417
Health					
Knows most fertile period during menstrual cycle,% [*n* = 1883]	1.6	0.4†	0.8	0.8	0.298
General self-efficacy score (0–6), mean (sd) [*n* = 1883]	2.3 (2.0)	2.3 (1.9)	2.2 (1.9)	1.7 (2.0)**	0.007
Wealth Creation					
Financial literacy score (0–10), mean (sd) [*n* = 1883]	4.5 (2.0)	4.2 (2.1)	4.1 (2.0)	4.1 (2.0)	0.571
Saved money in the past 6 months, % [*n* = 1883]	0.7	0.4	0.4	0.8	0.837
Household-level					
Household expects girl to complete secondary, % [*n* = 2008]	86.8	80.8	81.7	81.7	0.347
Household wealth quintile (1–5), mean (sd) [*n* = 2011]	3.0 (1.4)	2.7 (1.5)	2.9 (1.5)	2.9 (1.4)	0.791
Sample by arm when *n* = 1909	447	471	490	501	1909

Asterisks in columns 2–4 indicate statistically different from V-only. *P*-values in column 5 are from an F test for joint differences across study arms for sample of *N* = 1909 (unless otherwise noted) non-attritors in the two-year follow-up. All statistical tests carried out using robust standard errors allowing for clustering at the village level

*** *p* < 0.001, ** *p* < 0.01, * *p* < 0.05, † *p* < 0.1

We estimated linear probability models for each study site predicting re-interview in the two-year follow-up to examine its correlates and characterize potential attrition bias (Appendix Tables 4a and 4b). In addition to binary variables for the study arms, the models included baseline measures of age, cognitive test score, school enrollment (Wajir only), grade attainment, parental education, co-residence with parents, household wealth and district (Wajir only). Conditional on those covariates, in Kibera the probability of re-interview was nearly 10 percentage points higher in all three arms compared to V-only (Appendix Table 4a, column 1) and higher for the youngest, those with marginally higher cognitive test scores and those who resided with both of their parents. Expanding the model to also include interactions of the baseline controls with a binary variable for each study arm (column 2), however, indicates only one individually significant variable (paternal schooling) and neither the joint test of all interactions nor joint tests of interactions for each study arm are significant. Conditional on the covariates, in Wajir the probability of re-interview was about 4 percentage points higher in the VEH and VEHW arms compared to V-only (Appendix Table 4b, column 1), and higher for the youngest, those initially enrolled in school or with higher grade attainment, and those living in East Wajir. Expanding the model to also include interactions of the baseline controls with a binary variable for each study arm (column 2) indicates only a few significant differences between the relationship of the baseline controls and attrition. However, the joint test of all interactions and joint tests of interactions for the VEH and VEHW study arm are significant. The balance results across study arms and these patterns for attrition suggest that while there are some differences (especially in Wajir), large systematic biases in the ITT estimates threatening internal validity are unlikely. Nevertheless, to assess further the possibility of bias below we also characterize the results controlling for these additional covariates and also accounting for attrition using inverse probability weights.

At baseline in Kibera, girls averaged 12.6 years old and about half lived with their parents, a majority of whom had completed primary school. Nearly one-third of the girls had experienced violence perpetrated by a male in the past year, notably high given such violence is often underreported. Virtually all girls in Kibera were enrolled in school and average grade attainment was 5.7 years, 2 years below completed primary school and consistent with their ages. Over 90% were literate in Swahili and English and all but a few girls were expected to complete secondary school. Reproductive health knowledge was low, with fewer than one in ten girls able to identify the most fertile period during the menstrual cycle. Girls scored on average 4 out of 6 in general self-efficacy; many of the hypothetical situations posed in the scale proved difficult for younger girls to understand in both sites. On average girls scored nearly 6 out of 10 on financial literacy and about one-quarter had saved any money in the past 6 months.

At baseline in Wajir, girls averaged 11.9 years old and about three-quarters lived with their parents, very few of whom had ever themselves attended school. Girls were younger in Wajir on average largely because, unlike in Kibera, there was no time gap between the household listing when girls were screened by age and the baseline survey. In both settings some girls determined to be modestly outside the initial age ranges after initial inclusion, for example during the two-year follow-up survey, were retained in the samples (and all analyses control for age). Less than 5% experienced violence perpetrated by a male in the past year, although as in Kibera underreporting is possible. Approximately three-quarters of girls were enrolled in school and average grade attainment was only 2.9 years, about half that of their counterparts in Kibera. Low levels of schooling were reflected in literacy with less than 40% literate in both Swahili and English. Although lower than in Kibera, however, more than 80% of households still expected the girl to complete secondary school. Reproductive health knowledge and general self-efficacy were even lower in Wajir than in Kibera: hardly any girls were able to identify the most fertile period during the menstrual cycle, and they scored on average 2 out of 6 in general self-efficacy. On average girls scored 4 out of 10 in financial literacy, and virtually none had saved any money in the past 6 months.

Table 4 presents results from the ITT analyses for the Kibera study site. The first column displays the means of the two-year follow-up outcomes for the V-only study arm. The next three columns present the ITT estimates relative to V-only for the VE, VEH and VEHW study arms allowing assessment of the program effects on the secondary outcomes for each study arm. To explore whether there were incremental effects across study arms with additional interventions, in particular assessment of cash versus cash plus programming, the final three columns 5–7 present differences between pairs of study arms with the education intervention, based on the estimates in columns 2 to 4.

**Table 4:** Kibera estimated intent-to-treat effects at two-year follow-up, by study arm

	(1)	(2)	(3)	(4)	(5)	(6)	(7)
	V-Only two-year follow-up Mean	VE Estimate	VEH Estimate	VEHW Estimate	VEH vs VE (3)–(2)	VEHW vs VE (4)–(2)	VEHW vs VEH (4)–(3)
Violence Prevention							
Experienced violence by a male in the past year (=1)	0.422	− 0.088**	− 0.059*	− 0.042	0.028	0.046	0.018
95% CI		[− 0.14, − 0.03]	[− 0.12, 0.00]	[− 0.10, 0.02]	[− 0.03, 0.08]	[− 0.01, 0.10]	[− 0.04, 0.07]
Gender equitable attitudes z-score^a^	0.000	− 0.036	0.054	0.078	0.090	0.114*	0.024
95% CI		[−0.15, 0.08]	[− 0.07, 0.17]	[− 0.04, 0.19]	[− 0.02, 0.20]	[0.00, 0.23]	[− 0.09, 0.14]
Positive gender schooling attitudes z-score	0.211	−0.024	− 0.091†	− 0.137*	− 0.067	−0.113*	− 0.046
95% CI		[−0.12, 0.07]	[−0.19, 0.01]	[− 0.24, − 0.03]	[− 0.17, 0.03]	[− 0.22, − 0.01]	[− 0.15, 0.06]
Education							
Grade attainment	7.501	0.052†	0.048†	0.067*	− 0.003	0.016	0.019
95% CI		[0.00, 0.11]	[−0.01, 0.10]	[0.01, 0.12]	[−0.05, 0.05]	[−0.03, 0.06]	[− 0.03, 0.07]
Primary school complete (=1)	0.517	0.020	0.017	0.032	−0.004	0.011	0.015
95% CI		[−0.03, 0.07]	[− 0.03, 0.07]	[− 0.02, 0.08]	[− 0.05, 0.05]	[−0.04, 0.06]	[− 0.03, 0.06]
Enrolled in current school year (=1)	0.959	0.005	0.011	0.020†	0.006	0.015	0.010
95% CI		[−0.02, 0.03]	[−0.01, 0.03]	[0.00, 0.04]	[−0.01, 0.03]	[0.00, 0.03]	[−0.01, 0.03]
Conditional primary school complete (=1)^b^ [*n* = 1104]	0.887	0.037	0.020	0.070**	−0.016	0.033	0.050*
95% CI		[−0.01, 0.09]	[−0.03, 0.07]	[0.02, 0.12]	[−0.06, 0.03]	[− 0.01, 0.07]	[0.01, 0.09]
Conditional transition to secondary school (=1)^c^ [*n* = 1131]	0.836	0.036	0.035	0.071*	−0.001	0.036	0.037
95% CI		[−0.02, 0.10]	[−0.02, 0.09]	[0.01, 0.13]	[−0.06, 0.05]	[− 0.02, 0.09]	[− 0.01, 0.09]
Health							
Knows most fertile period during menstrual cycle (=1)	0.087	0.016	0.019	0.022	0.003	0.006	0.003
95% CI		[−0.02, 0.05]	[−0.02, 0.05]	[− 0.01, 0.06]	[− 0.03, 0.04]	[−0.03, 0.04]	[− 0.03, 0.04]
Knows method of modern contraception^a^ (=1) [*n* = 2175]	0.555	−0.007	0.130***	0.119***	0.137***	0.125***	−0.011
95% CI		[− 0.06, 0.05]	[0.07, 0.19]	[0.06, 0.18]	[0.08, 0.19]	[0.07, 0.18]	[−0.06, 0.04]
SRH knowledge z-score^a^ [*n* = 1948]	0.000	0.016	0.213**	0.158*	0.197**	0.142*	−0.055
95% CI		[−0.11, 0.14]	[0.09, 0.34]	[0.03, 0.29]	[0.08, 0.31]	[0.02, 0.26]	[−0.17, 0.07]
General self-efficacy z-score	0.325	0.152**	0.030	0.055	−0.122*	−0.097*	0.025
95% CI		[0.06, 0.25]	[−0.07, 0.13]	[−0.05, 0.16]	[− 0.22, − 0.03]	[−0.19, 0.00]	[− 0.08, 0.12]
Condom use self-efficacy z-score^a^ [*n* = 1777]	0.000	0.038	0.178**	0.124†	0.140*	0.086	−0.054
95% CI		[−0.10, 0.17]	[0.05, 0.30]	[−0.01, 0.25]	[0.02, 0.26]	[−0.04, 0.21]	[−0.17, 0.06]
Wealth creation							
Financial literacy z-score	−0.085	0.014	0.045	0.381***	0.030	0.366***	0.336***
95% CI		[−0.11, 0.13]	[−0.07, 0.16]	[0.26, 0.50]	[−0.09, 0.15]	[0.25, 0.48]	[0.22, 0.45]
Saved money in the past 6 months (=1)	0.448	0.000	0.017	0.202***	0.017	0.202***	0.185***
95% CI		[−0.06, 0.06]	[− 0.04, 0.08]	[0.14, 0.26]	[− 0.04, 0.07]	[0.15, 0.26]	[0.13, 0.24]
Household-level outcomes							
Expects girl to complete secondary school (=1) [*n* = 2193]	0.994	0.002	−0.001	0.004	−0.003	0.002	0.005
95% CI		[−0.01, 0.01]	[−0.01, 0.01]	[0.00, 0.01]	[− 0.01, 0.01]	[0.00, 0.01]	[0.00, 0.01]
Household wealth quintile [*n* = 2236]	2.823	0.101	0.123	0.105	0.022	0.004	−0.019
95% CI		[−0.06, 0.26]	[−0.03, 0.28]	[− 0.06, 0.27]	[− 0.13, 0.18]	[− 0.15, 0.16]	[− 0.17, 0.14]
Summary index z-scores							
*Violence prevention outcomes summary index z-score*^d^	0.000	0.066	0.037	−0.004	− 0.029	− 0.070	−0.040
95% CI		[−0.05, 0.19]	[−0.08, 0.16]	[− 0.13, 0.12]	[− 0.15, 0.09]	[− 0.19, 0.05]	[− 0.16, 0.08]
*Education outcomes summary index z-score (grade, primary, enroll)*	0.000	0.062	0.082	0.123*	0.020	0.061	0.041
95% CI		[−0.04, 0.17]	[− 0.02, 0.18]	[0.03, 0.22]	[−0.07, 0.12]	[− 0.03, 0.15]	[− 0.05, 0.13]
*Health outcomes summary index z-score*	0.000	0.113†	0.306***	0.279***	0.193**	0.167**	−0.026
95% CI		[0.00, 0.23]	[0.19, 0.42]	[0.16, 0.40]	[0.08, 0.31]	[0.05, 0.28]	[−0.14, 0.09]
*Wealth creation outcomes summary index z-score*	0.000	0.015	0.056	0.517***	0.041	0.502***	0.461***
95% CI		[−0.10, 0.13]	[− 0.06, 0.17]	[0.40, 0.63]	[−0.07, 0.16]	[0.39, 0.62]	[0.35, 0.57]

The table reports two-year follow-up means for V-only in column 1, the OLS estimated ITT effect for each study arm relative to V-only in columns 2–4 and differences in the estimated ITT effects across study arms in columns 5–7. For binary outcomes (=1) these are linear probability models. Column 5 compares the estimates for VEH to VE, column 6 compares VEHW to VE, and column 7 compares VEHW to VEH. For example, the estimate in column 7 for ‘Experienced violence by a male in the past year’ (0.018) is the difference between the estimate for VEHW (− 0.042) in column 4 and the estimate for VEH (− 0.059) in column 3. Minor differences in the reported differentials compared to the estimates presented in columns 2–4 are due to rounding. Numbers in square brackets indicate 95% confidence intervals. Regressions were estimated with robust standard errors and included controls for age and the outcome measured at baseline unless otherwise noted. *N* = 2190; sample is smaller for some individual outcomes due to missing data as indicated

*** *p* < 0.001, ** *p* < 0.01, * *p* < 0.05, † *p* < 0.1

^a^ No baseline control for outcome variable available

^b^ Among girls who had completed grade 6 but had not yet completed grade 8 at baseline

^c^ Among girls who had completed grade 6 but had not yet enrolled in secondary school at baseline

^d^ Violence prevention variable is reverse coded prior to inclusion in summary variable

The intervention led to reductions in the experience of male-perpetrated violence between 4 and 9 percentage points compared with an average of 42% in the V-only arm, statistically significant for the VE and VEH arms, although VEH is not significant after consideration of multiple hypothesis testing based on the FDR q-value (Appendix Table 5). There was a modest reduction in gender schooling attitudes of about 0.1 SD in VEHW, but no effect on overall gender equitable attitudes or improvements in the violence prevention summary z-score (Table 5, bottom panel). For schooling, there were small positive effects on grade attainment of about 0.05 grades (only significant for VEHW) and small positive but insignificant effects on primary school completion and enrollment. Examining these three variables together in a summary variable suggests an increase of about 0.1 SD for the VEHW arm only. Primary school in Kenya consists of eight grades and a critical bottleneck in schooling is advancing from primary to secondary school. Therefore in Kibera, where schooling attainment was substantially higher, in addition to commonly studied highest grade completed and current enrollment [65] we also considered two conditional outcomes examining progression during that transition. Consideration of the conditional schooling variables indicates VEHW increased the probability of completing primary school and, separately, transitioning to secondary school by 7 percentage points. Positive and statistically significant effects are seen for the VEH and VEHW arms in SRH knowledge outcomes and condom self-efficacy. There was a greater than 10 percentage point increase in knowledge of at least one method of modern contraception compared with 56% knowledge in V-only, and a 0.2 SD increase in general SRH knowledge and more than 0.1 SD increase in condom self-efficacy. A positive and statistically significant effect of more than 0.1 SD is observed for general self-efficacy in the VE arm. Estimation of the impact on the summary health outcome average z-score yields significant increases of about 0.3 SD for the VEH and VEHW study arms. Large positive and statistically significant effects on both financial literacy and saving behavior are found for the VEHW arm (and correspondingly in the summary measure), but not elsewhere. For example, savings increased by 20 percentage points compared with 45% in V-only. There were no effects of the interventions on the household-level wealth quintile and, unsurprisingly given the near-100% levels at baseline, no effects on household expectations the girl would complete secondary school.

**Table 5:** Wajir estimated intent-to-treat effects at two-year follow-up, by study arm

	(1)	(2)	(3)	(4)	(5)	(6)	(7)
	V-Only two-year follow-up Mean	VE Estimate	VEH Estimate	VEHW Estimate	VEH vs VE (3)–(2)	VEHW vs VE (4)–(2)	VEHW vs VEH (4)–(3)
Violence Prevention							
Experienced violence by a male in the past year (=1) [*n* = 1878]	0.038	−0.006	0.015	− 0.022	0.021	−0.017	− 0.037*
95% CI		[−0.04, 0.03]	[−0.02, 0.05]	[− 0.05, 0.01]	[− 0.02, 0.06]	[− 0.05, 0.01]	[− 0.07, − 0.01]
Gender equitable attitudes z-score^a^ [*n* = 1903]	0.000	−0.171	− 0.208*	− 0.018	− 0.037	0.153	0.190†
95% CI		[−0.39, 0.05]	[−0.39, − 0.02]	[− 0.26, 0.22]	[− 0.23, 0.15]	[− 0.09, 0.39]	[− 0.02, 0.40]
Positive gender schooling attitudes z-score [*n* = 1878]	0.160	0.156*	0.099	0.068	−0.057	−0.088	− 0.031
95% CI		[0.00, 0.31]	[−0.05, 0.25]	[− 0.08, 0.21]	[− 0.22, 0.10]	[− 0.23, 0.06]	[− 0.18, 0.12]
Education							
Grade attainment	4.492	0.259**	0.194*	0.181*	−0.065	− 0.079	− 0.013
95% CI		[0.10, 0.42]	[0.02, 0.36]	[0.01, 0.35]	[−0.23, 0.10]	[− 0.24, 0.09]	[− 0.19, 0.17]
Primary school complete (=1)	0.130	0.010	0.021	−0.029	0.011	−0.039	− 0.050
95% CI		[−0.05, 0.07]	[−0.05, 0.09]	[− 0.08, 0.03]	[− 0.06, 0.08]	[− 0.10, 0.02]	[− 0.11, 0.01]
Enrolled in current school year (=1)	0.808	0.144***	0.070*	0.084**	−0.074*	−0.060*	0.014
95% CI		[0.09, 0.20]	[0.01, 0.13]	[0.02, 0.14]	[−0.13, − 0.02]	[− 0.11, − 0.01]	[−0.05, 0.08]
Health							
Knows most fertile period during menstrual cycle (=1) [*n* = 1878]	0.049	−0.031†	−0.021	− 0.013	0.010	0.018	0.008
95% CI		[−0.06, 0.00]	[−0.05, 0.01]	[− 0.04, 0.02]	[− 0.01, 0.03]	[−0.01, 0.04]	[− 0.01, 0.03]
Knows method of modern contraception^a^ (=1) [*n* = 1848]	0.390	−0.097†	− 0.103*	− 0.096†	− 0.007	0.001	0.007
95% CI		[−0.21, 0.02]	[−0.19, − 0.01]	[−0.20, 0.01]	[− 0.10, 0.09]	[− 0.11, 0.11]	[−0.08, 0.10]
SRH knowledge z-score^a,b^ [*n* = 1400]	0.000	0.344*	0.433**	0.234†	0.090	−0.110	−0.199
95% CI		[0.08, 0.60]	[0.17, 0.70]	[−0.03, 0.50]	[−0.17, 0.35]	[− 0.37, 0.15]	[− 0.47, 0.07]
General self-efficacy z-score [*n* = 1878]	0.990	− 0.054	−0.014	− 0.030	0.040	0.024	−0.016
95% CI		[−0.25, 0.14]	[−0.22, 0.19]	[− 0.22, 0.16]	[− 0.17, 0.25]	[−0.17, 0.21]	[− 0.21, 0.18]
Wealth creation							
Financial literacy z-score [*n* = 1878]	0.439	−0.069	0.030	0.185	0.099	0.254*	0.155
95% CI		[−0.31, 0.17]	[−0.19, 0.25]	[− 0.06, 0.43]	[− 0.08, 0.28]	[0.04, 0.46]	[− 0.04, 0.35]
Saved money in the past 6 months (=1) [*n* = 1878]	0.011	0.030	0.047*	0.409***	0.017	0.379***	0.362***
95% CI		[−0.01, 0.07]	[0.01, 0.09]	[0.31, 0.51]	[−0.03, 0.06]	[0.27, 0.48]	[0.26, 0.47]
Household-level outcomes							
Expects girl to complete secondary school (=1) [*n* = 2007]	0.861	0.058*	0.050†	0.043	−0.008	−0.015	−0.007
95% CI		[0.01, 0.10]	[0.00, 0.10]	[−0.01, 0.10]	[−0.05, 0.04]	[− 0.06, 0.03]	[− 0.06, 0.05]
Household wealth quintile [*n* = 2011]	3.226	− 0.434*	−0.247	− 0.211	0.188	0.223	0.035
95% CI		[−0.83, − 0.04]	[− 0.64, 0.14]	[−0.54, 0.12]	[− 0.25, 0.62]	[− 0.16, 0.60]	[−0.34, 0.41]
Summary index z-scores							
*Violence prevention outcomes summary index z-score*^*c*^ [*n* = 1878]	0.000	0.030	−0.101	0.104	−0.131†	0.073	0.205**
95% CI		[−0.15, 0.21]	[−0.29, 0.08]	[− 0.07, 0.27]	[− 0.29, 0.02]	[−0.06, 0.21]	[0.06, 0.35]
*Education outcomes summary index z-score*	0.000	0.302***	0.189*	0.122	−0.114	−0.180*	− 0.067
95% CI		[0.16, 0.44]	[0.02, 0.36]	[−0.03, 0.27]	[−0.29, 0.07]	[− 0.33, − 0.03]	[−0.25, 0.12]
*Health outcomes summary index z-score* [*n* = 1878]	0.000	−0.026	0.066	−0.014	0.092	0.012	−0.080
95% CI		[−0.22, 0.17]	[−0.13, 0.26]	[− 0.22, 0.19]	[− 0.06, 0.24]	[−0.15, 0.17]	[− 0.24, 0.08]
*Wealth creation outcomes summary index z-score* [*n* = 1878]	0.000	0.167	0.349*	2.852***	0.182	2.686***	2.504***
95% CI		[−0.14, 0.47]	[0.03, 0.66]	[2.14, 3.56]	[−0.15, 0.51]	[1.97, 3.40]	[1.79, 3.22]

The table reports two-year follow-up means for V-only in column 1, the OLS estimated ITT effect for each study arm relative to V-only in columns 2–4 and differences in the estimated ITT effects across study arms in columns 5–7. For binary outcomes (=1) these are linear probability models. Column 5 compares the estimates for VEH to VE, column 6 compares VEHW to VE, and column 7 compares VEHW to VEH. For example, the estimate in column 7 for ‘Experienced violence by a male in the past year’ (−0.037) is the difference between the estimate for VEHW (− 0.022) in column 4 and the estimate for VEH (0.015) in column 3. Minor differences in the reported differentials compared to the estimates presented in columns 2–4 are due to rounding. Numbers in square brackets indicate 95% confidence intervals. Regressions were estimated with standard errors clustered at the village level and included controls for 2009 district per the stratified randomization, age and the outcome measured at baseline unless otherwise noted. *N* = 1909; sample is smaller for some individual outcomes due to missing data as indicated

*** *p* < 0.001, ** *p* < 0.01, * *p* < 0.05, † *p* < 0.1

^a^ No baseline control for outcome variable available

^b^ Non-response for one or more items on the scale ranged from 24 to 30% across study arms and was higher for younger girls

^c^ Violence prevention variable is reverse coded prior to inclusion in summary variable

All results are robust to the inclusion of additional controls and to weighting for attrition, with significant point estimates changing only marginally and remaining significant at a 0.05 level (Appendix Table 5). The results for summary variables are also robust to the different specifications (Appendix Table 6). In addition, consideration of the combined education treatments (Appendix Table 6, column 5) indicate for all but the violence prevention domain positive significant effects of nearly 0.1 SD for education and 0.2 SD for health and wealth creation. Finally, results for all binary variable outcomes are the same in sign and significance based on odds ratios estimated via logistic regression (Appendix Table 7).

Examining columns 5–7 in Table 4, there were few significant differences between the effects of study arms with the education intervention for violence prevention and education outcomes. Several health variables, including the summary variable, were significantly larger in the VEH and VEHW arms compared with the VE arm. One notable exception is general self-efficacy which showed improvement only in the VE study arm. The two wealth creation variables (and the summary measure) were larger in the VEHW compared with the VE and VEH arms.

Table 5 presents parallel results from the ITT analyses for the Wajir study site. There were few effects on outcomes related to violence prevention and after accounting for multiple hypothesis testing no significant effects (Appendix Table 7) which was unsurprising given the research design. There were large significant effects on schooling, approximately 0.2 additional grades and increased enrollment of 7–14 percentage points compared to an enrollment rate of 80% in V-only. This improvement is on par with some of the most effective CCT programs for schooling elsewhere [65]. There was no increase, however, in completed primary school, likely reflecting the initial low levels of schooling for most girls at the start of the intervention. The point estimates for effects on health outcomes suggest mixed results, although none of the negative estimates for knowing the most fertile period or a modern method of contraception are significant after accounting for multiple hypothesis testing. There were substantial significant improvements for sexual and reproductive knowledge of 0.3 SD or more for VE and VEH. There were no effects on financial literacy but positive and statistically significant effects for the VEH (not significant after accounting for multiple hypothesis testing) and VEHW arms in savings behavior, and the effect for VEHW was a substantial 40 percentage point increase on a base of under 2% in V-only. At the household level, there was an apparent unexpected decline in wealth quintile for the VE study arm, not significant after accounting for multiple hypothesis testing. A robust significant increase of about 5 percentage points (compared with 86% in V-only) was seen for household expectations the girl would complete secondary school for the VE study arm.

Significant results are robust to the inclusion of additional controls and to weighting for attrition, with point estimates and significance levels only changing marginally for the majority of outcomes (Appendix Table 7). The results for summary variables are also robust to the different specifications (Appendix Table 8). In addition, consideration of the combined education treatments (Appendix Table 8, column 5) indicates positive significant effects of 0.2 SD for education and more than 1 SD for wealth creation. Finally, results for all binary variable outcomes are the same in sign and similar in significance based on odds ratios estimated via logistic regression (Appendix Table 10).

There are only a few significantly different effects between study arms with the education intervention in Wajir in columns 5–7 of Table 5. There was a significant difference between VEHW and VEH in violence experienced by a male, mirrored by a significant difference between the study arms in the violence prevention summary measure. Reflecting impacts on enrollment in the VE arm twice the size of the others, both VEH and VEHW had significantly lower impacts on enrollment (6–7 percentage points). Effects on financial literacy and savings behavior were substantially larger in the VEHW arm compared to the other two study arms.

## Discussion

Taken together, the results support the hypothesis that a multisectoral approach is needed to improve different outcomes important to the well-being of young adolescent girls across multiple domains, including education, health, and financial practices. Focusing on the summary variables, in Kibera VEH improved only the health summary, whereas VEHW improved the education, health and wealth creation summary variables. In Wajir, VE and VEH improved education and VEHW improved wealth creation. Hence for the most part the sector-specific intervention components directly contributed to the outcomes within their domain (or the summary measure) with fewer impacts on specific sector outcomes in study arms without the corresponding sector intervention. For example, education conditional cash transfers alone (the VE study arm) had no or only small effects on the health and wealth creation outcomes examined. Rather, the education intervention improved educational outcomes, the health intervention increased health knowledge (in Kibera only) and the wealth creation intervention increased financial literacy and savings behavior. Longer-term follow up will determine whether improvements in these intermediate outcomes will delay childbearing, the primary outcome of the AGI-K study.

This paper contributes to the literature on cash versus cash-plus programming. The empowerment components of the intervention (girl group sessions following the HLS and financial literacy curricula) led to positive changes for a subset of outcomes that cash alone did not influence. The findings are consistent with related literature that cash alone is not a “magic bullet” because it does not influence some potentially important outcomes [43], although it remains a key component of the package of interventions needed to address the array of challenges facing adolescents during their transition to adulthood. The cash transfers appear to have been critical in this context in part because they addressed the economic constraints at the household level, while also incentivizing education for the girls. This is in line with other studies in which girls empowerment programming took place without an education cash transfer and for which there were neither short nor long-term benefits on school enrollment [19]. Therefore, policy makers and program implementers may consider coupling cash transfer programs meant to improve adolescent programs with additional activities aimed at improving knowledge and skills in relevant sectors such as health or financial literacy.

The two distinct sites also provide new examples in which context and cultural norms moderate the effectiveness of programs, even after they have been carefully tailored to different settings. While in general impacts at a sectoral level were similar in both Kibera and Wajir, suggesting a degree of external validity of the results to different contexts, the specific variables affected differed across the sites. For example, in Kibera the interventions did not improve enrollment but in VEHW improved primary school completion and the transition to secondary school for girls who had been in their final 2 years of primary school at baseline, whereas in Wajir the programs largely worked to get girls who were not in school at baseline to enroll, in most cases for the first time. This is almost certainly due to the differences in schooling at baseline—with 99% of girls enrolled and 72% studying at the appropriate grade level for their age in Kibera compared to only 73 and 24% in Wajir. Only in Wajir was there an increase in the household expectation the girl would complete secondary school, since at baseline expectations were nearly 100% in Kibera. In both settings, the estimated effects on educational outcomes were often modestly smaller in VEH and VEHW compared to VE, possibly due to the additional complexity and burden of implementing and participating in those arms relative to VE. This points to a possible trade-off between single-sector programs focused on achieving the largest impacts for one specific outcome versus more complex programs that can achieve results across a wider set of outcomes, albeit with possibly smaller effect sizes. The health intervention led to SRH knowledge increases in Kibera, but not in Wajir. We hypothesize that there were not clearer gains in knowledge in Wajir possibly because of the inexperience of mentors or discomfort they felt when delivering SRH content even with the modified audio sessions, particularly around contraception since among Muslim Somalis it is culturally inappropriate to discuss fertility or contraception with unmarried females [66]. Context also may have important implications for the longer-term outcomes related to these interventions, and it is likely that differing context and cultural norms will continue to influence the pathways through which other outcomes are achieved, particularly with respect to eventual marriage and fertility decisions.

This paper has several limitations. The main limitation is that there is no randomized control and we cannot isolate the effect of the violence prevention intervention. Although violence prevention broadly conceived is critical in the socioecological model [18] and to the theory of change underpinning the programs, we are unable to assess empirically how important it was to include a community-level intervention focused on changing attitudes and norms to increase the value of girls or reduce violence toward them. At the same time, if the violence prevention intervention had beneficial effects on any of the outcomes the reported results are plausibly attenuated and therefore conservative estimates of the overall program impacts. Second, we did not implement the health or wealth creation interventions on their own or together without the education intervention, so it is not possible to assess the effectiveness of programming only focused on empowering the girls with the education conditional cash transfers. Third, girls randomized to the health and wealth interventions which included attendance at weekly meetings had different levels of participation and thus different exposures to the interventions; all estimates presented are ITT. Fourth, in Kibera (but not Wajir because of the distances between clusters) there was the possibility of spillover effects between girls randomized to different arms but living near one another and overlap of girls in each study arm within the same schools was proportional to the randomization. Because enrollment was nearly universal at baseline, however, there were only small increases in enrollment so it was unlikely the interventions substantively increased school crowding or depressed enrollment for girls in the V-only arm. On the other hand, although the bulk of the transfers were delivered to the schools directly, girls receiving transfers could share resources and information with those not receiving them or serve as positive role models, leading to positive spillovers and consequently more conservative point estimates of program impact. Finally, as the girls in the sample were still relatively young at the two-year follow-up, we do not have the statistical power to test the impact on longer-term outcomes such as secondary school completion, timing of sexual debut, first pregnancy or marriage. This limitation also means we do not consider cost-effectiveness of the interventions as they would necessarily be only partial in nature at this timepoint. These outcomes will be the focus using data collected 2 years after the end of the intervention.

Offsetting these limitations, this paper has several strengths, including a randomized research design implemented in two different settings, high participation with virtually no program contamination in the form of girls receiving interventions they were not randomized to, high longitudinal follow-up rates and measurements on both the adolescent girl and her household.

## Conclusions

This paper contributes to the growing literature on multisectoral programming for adolescent girls and the cash versus cash plus debate. The findings reinforce the premise that addressing empowerment for adolescent girls through a multisectoral approach can lead to broader impacts and that cash plus, i.e., supplementing economic resources for the household with social, health and asset-building skills for the girls themselves, can provide a wider range of beneficial impacts for them across education, health and economic outcomes. Program and policy design should consider opportunities to leverage or combine social protection at the household level with individual programs for adolescent girls to increase overall benefits for the girls.

## Supplementary Information

**Additional file 1 : Appendix Table.** Key Variables for AGI-K Primary and Secondary Outcomes. **Appendix Table 2.** Kibera baseline means of key variables for all observations, by study arm. **Appendix Table 3.** Wajir baseline means of key variables for all observations, by study arm. **Appendix Table 4.** Correlates of two-year follow-up survey response. **Appendix Table 5.** Kibera estimated ITT effects, additional results for individual outcomes. **Appendix Table 6.** Kibera estimated ITT effects, additional results for summary measures. **Appendix Table 7.** Kibera estimated ITT odds ratio effects for binary outcomes**. Appendix Table 8.** Wajir estimated ITT effects, additional results for individual outcomes. **Appendix Table 9.** Wajir estimated ITT effects, additional results for summary measures. **Appendix Table 10.** Wajir estimated ITT odds ratio effects for binary outcomes**. Appendix Table 11.** Two-year follow-up outcomes variable definitions.

## Data Availability

Study data in this paper, including de-identified individual data and data dictionary, will be made available open access upon publication. The data will be stored and available for downloading via the Adolescent Data Hub - http://popcouncil.org/girlcenter/adolescentdatahub/.

## Abbreviations

AGI-K: Adolescent Girls Initiative – Kenya
ANCOVA: Analysis of Co-Variance
CCT: Conditional Cash Transfer
FDR: False Discovery Rate
FE: Financial Education
FGM/C: Female Genital Mutilation/Cutting
ITT: Intent-to-Treat
KDHS: Kenya Demographic and Health Survey
NCSSS: Nairobi Cross-Sectional Slum Survey
PCA: Principal Components Analysis
NGO: Non-governmental Organization
SD: Standard Deviation
SRH: Sexual and Reproductive Health
