# Supplementary material for: Impacts of two-year multisectoral cash plus programs on young adolescent girls’ education, health and economic outcomes: Adolescent Girls Initiative-Kenya (AGI-K) randomized trial
Source: BMC Public Health. 2021 Nov 24;21:2159. doi: 10.1186/s12889-021-12224-3 (PMC8613919; doi:10.1186/s12889-021-12224-3)
Supplement: Supplementary file 1 — Additional file 1 : Appendix Table. Key Variables for AGI-K Primary and Secondary Outcomes. Appendix Table 2. Kibera baseline means of key variables for all observations, by study arm. Appendix Table 3. Wajir baseline means of key variables for all observations, by study arm. Appendix Table 4. Correlates of two-year follow-up survey response. Appendix Table 5. Kibera estimated ITT effects, additional results for individual outcomes. Appendix Table 6. Kibera estimated ITT effects, additional results for summary measures. Appendix Table 7. Kibera estimated ITT odds ratio effects for binary outcomes. Appendix Table 8. Wajir estimated ITT effects, additional results for individual outcomes. Appendix Table 9. Wajir estimated ITT effects, additional results for summary measures. Appendix Table 10. Wajir estimated ITT odds ratio effects for binary outcomes. Appendix Table 11. Two-year follow-up outcomes variable definitions. [file 12889_2021_12224_MOESM1_ESM.docx]

Online Appendices for

**Impacts of two-year multisectoral cash plus programs on young adolescent girls’ education, health and economic outcomes: Adolescent Girls Initiative-Kenya (AGI-K) randomized trial**

Karen Austrian, Erica Soler-Hampejsek, Beth Kangwana, Yohannes Dibaba Wado, Benta Abuya and John A. Maluccio

Appendix Table 1: Key Variables for AGI-K Primary and Secondary Outcomes

Appendix Table 2: Kibera baseline means of key variables for all observations, by study arm

Appendix Table 3: Wajir baseline means of key variables for all observations, by study arm

Appendix Table 4: Correlates of two-year follow-up survey response

Appendix Table 5: Kibera estimated ITT effects, additional results for individual outcomes

Appendix Table 6: Kibera estimated ITT effects, additional results for summary measures

Appendix Table 7: Kibera estimated ITT odds ratio effects for binary outcomes

Appendix Table 8: Wajir estimated ITT effects, additional results for individual outcomes

Appendix Table 9: Wajir estimated ITT effects, additional results for summary measures

Appendix Table 10: Wajir estimated ITT odds ratio effects for binary outcomes

Appendix Table 11: Two-year follow-up outcomes variable definitions

**Appendix Table 1. Key Variables and Expected Effects for AGI-K Primary and Secondary Outcomes**

| PRIMARY OUTCOMES | | | | |
| --- | --- | --- | --- | --- |
| WELL-BEING | Age at first birth (+) | Age at first sex (+) | | Age at marriage (+) |
| SECONDARY OUTCOMES | | | | |
| VIOLENCE | Experience of gender-based violence (-) | Positive gender norms related to violence (+) |  | |
| EDUCATION | Mean grade of schooling (+) | Rate of primary school completion (+) |  | |
| HEALTH | Knowledge on sexual and reproductive health (+) | Decision-making skills (+) | | Contraceptive use (+) |
| WEALTH | Knowledge on financial education (+) | Saving (+) | | Participation in income generating activities (+)^1^ |

Source: ISRCTN Trial registry (<http://www.isrctn.com/ISRCTN77455458>) first outlined in Institutional Review Board application to the Population Council and to the Kenyan AMREF Ethics and Scientific Review Committee, both approved in 2014.

^1^ Participation in income generating activities was excluded from the analyses for the two-year follow-up when girls were still relatively young for participation in the labor market to be considered beneficial. At baseline, only 11% of girls reported working for pay in the last year in Kibera and even fewer in Wajir, likely due to their young ages (appendix Tables 2 and 3).

**Appendix** **Table 2. Kibera baseline means of key variables for all observations, by study arm**

|  | (1) | | (2) | (3) | (4) | (5) |
| --- | --- | --- | --- | --- | --- | --- |
|  | V-Only | | VE | VEH | VEHW | Overall |
| Background |  | |  |  |  |  |
| Age, mean (sd) | 12.6 (1.2) | | 12.5 (1.3) | 12.6 (1.2) | 12.5 (1.3) | 12.6 (1.3) |
| Cognitive score (0–16), mean (sd) [2,373] | 8.2 (3.0) | | 8.4 (3.0) | 8.3 (3.1) | 8.3 (3.2) | 8.3 (3.1) |
| Lives with both parents (=1), % [n=2,373] | 52.1 | | 55.0 | 50.0 | 52.6 | 52.4 |
| Mother completed primary school (=1), % [n=2,227] | 63.0 | | 62.7 | 62.7 | 64.5 | 63.2 |
| Father completed primary school (=1), % [n=1,962] | 76.3 | | 79.3 | 75.3 | 79.5 | 77.6 |
| Violence Prevention |  | |  |  |  |  |
| Experienced violence by a male in the past year (=1), % | 29.3 | | 29.9 | 31.4 | 33.1 | 30.9 |
| Positive gender schooling attitudes score (0-4), mean (sd) | 3.6 (0.7) | | 3.6 (0.7) | 3.6 (0.7) | 3.6 (0.7) | 3.6 (0.7) |
| Education |  | |  |  |  |  |
| Grade attainment, mean (sd) | 5.7 (1.4) | | 5.7 (1.3) | 5.7 (1.4) | 5.7 (1.3) | 5.7 (1.4) |
| Primary school complete (=1), % | 8.0 | | 5.9 | 6.7 | 6.1 | 6.7 |
| Enrolled in school (=1), % | 99.3 | | 99.2 | 98.4 | 99.2 | 99.0 |
| Literate in Swahili and English (=1), % [n=2,373] | 91.9 | | 93.4 | 92.9 | 93.4 | 92.9 |
| Health |  | |  |  |  |  |
| Knows the most fertile period during menstrual cycle (=1), % | 8.4 | | 8.3 | 7.1 | 6.1 | 7.5 |
| General self-efficacy score (0-6), mean (sd) | 3.8 (1.7) | | 4.0 (1.6) | 3.9 (1.6) | 4.0 (1.6) | 3.9 (1.6) |
| Wealth Creation |  | |  |  |  |  |
| Financial literacy score (0-10), mean (sd) | 5.7 (1.9) | | 5.6 (1.9) | 5.6 (1.9) | 5.8 (1.9) | 5.7 (1.9) |
| Saved money in the past six months (=1), % | 27.1 | | 25.2 | 26.4 | 28.4 | 26.8 |
| Worked for pay in the last year (=1), % | 10.9 | | 12.0 | 11.7 | 10.0 | 11.1 |
| Household-level |  | |  |  |  |  |
| Household expects girl to complete secondary (=1), % [n=2,358] | | 99.8 | 99.7 | 99.7 | 99.8 | 99.7 |
| Household expected girl to marry before age 22 (=1),^1^ % [n=1,874] | | 2.6 | 4.1 | 3.4 | 4.7 | 3.7 |
| Household wealth quintile (1-5), mean (sd) [n=2,374] | 3.1 (1.4) | | 3.0 (1.4) | 3.0 (1.4) | 3.0 (1.4) | 3.0 (1.4) |
| Sample by arm when n=2,390 | 597 | | 592 | 609 | 592 | 2,390 |

Notes: N = 2,390 unless otherwise indicated. Final column is overall average. ^1^ Excludes “do not know” responses.

**Appendix** **Table 3. Wajir baseline means of key variables for all observations, by study arm**

|  | (1) | | | (2) | (3) | (4) | (5) |
| --- | --- | --- | --- | --- | --- | --- | --- |
|  | V-Only | | | VE | VEH | VEHW | Overall |
| Background |  | | |  |  |  |  |
| Age, mean (sd) | 11.9 (1.3) | | | 12.0 (1.3) | 11.8 (1.2) | 11.9 (1.3) | 11.9 (1.3) |
| Cognitive score (0–16), mean (sd) [2,109] | 5.2 (2.9) | | | 5.0 (3.0) | 4.9 (3.2) | 5.3 (3.1) | 5.1 (3.0) |
| Lives with both parents (=1), % [n=2,124] | 73.5 | | | 73.0 | 76.9 | 73.8 | 74.3 |
| Mother ever attended school (=1), % [n=2,123] | 1.2 | | | 1.3 | 0.6 | 0.4 | 0.8 |
| Father ever attended school (=1), % [n=2,120] | 5.6 | | | 2.7 | 3.2 | 3.3 | 3.7 |
| Violence Prevention |  | | |  |  |  |  |
| Experienced violence by a male in the past year (=1), % [n=2,121] | | 4.0 | | 4.1 | 3.0 | 2.2 | 3.3 |
| Positive gender schooling attitudes (0-4), mean (sd) [n=2,121] | 3.0 (1.0) | | | 2.8 (1.0) | 2.9 (1.1) | 2.9 (1.1) | 2.9 (1.1) |
| Education |  | | |  |  |  |  |
| Grade attainment, mean (sd) | 2.9 (2.2) | | | 2.7 (2.3) | 2.7 (2.2) | 2.5 (2.1) | 2.7 (2.2) |
| Primary school complete (=1), % | 1.2 | | | 0.9 | 0.7 | 0.7 | 0.9 |
| Enrolled in school (=1), % | 78.5 | | | 68.2 | 75.0 | 70.6 | 72.9 |
| Literate in Swahili and English (=1), % [n=2,109] | 38.0 | | | 31.9 | 37.3 | 32.6 | 34.9 |
| Health |  | | |  |  |  |  |
| Knows the most fertile period during menstrual cycle (=1), % [2,121] | | | 1.4 | 0.6 | 0.8 | 0.7 | 0.8 |
| General self-efficacy score (0-6), mean (sd) [2,121] | 2.3 (2.0) | | | 2.3 (1.9) | 2.2 (1.9) | 1.7 (2.0) | 2.1 (2.0) |
| Wealth Creation |  | | |  |  |  |  |
| Financial literacy score (0-10), mean (sd) [2,121] | 4.4 (2.0) | | | 4.1 (2.1) | 4.0 (2.0) | 4.0 (2.0) | 4.1 (2.0) |
| Saved money in the past six months (=1), % [2,121] | 0.6 | | | 0.4 | 0.4 | 0.7 | 0.5 |
| Worked for pay in the last year (=1), % [2,121] | 5.2 | | | 5.7 | 3.6 | 3.5 | 4.5 |
| Household-level |  | | |  |  |  |  |
| Household expects girl to complete secondary (=1), % [n=2,119] | | 85.8 | | 80.0 | 80.9 | 81.7 | 82.0 |
| Household expected girl to marry before age 18 (=1),^1^ % [n=777] | | 2.5 | | 2.9 | 8.1 | 5.7 | 4.9 |
| Household wealth quintile (1-5), mean (sd) [n=2,123] | 3.0 (1.4) | | | 2.8 (1.5) | 3.0 (1.5) | 2.9 (1.4) | 2.9 (1.5) |
| Sample by arm when n=2,147 | 506 | | | 553 | 537 | 551 | 2,147 |

Notes: N = 2,147 unless otherwise indicated. Final column is overall average. ^1^ Excludes “do not know” responses.

**Appendix Table 4a. Kibera correlates of two-year follow-up survey response, by study arm**

| Dependent variable: Interviewed at two-year follow-up (=1) | | (1) | (2a) | (2b) | (2c) | (2d) |
| --- | --- | --- | --- | --- | --- | --- |
|  |  | | X | Study arm 2*X | Study arm 3*X | Study arm 4*X |
| Study arms: V-only (ref) |  | |  |  |  |  |
| VE (=1) | 0.096*** | | 0.186 |  |  |  |
|  | (0.017) | | (0.165) |  |  |  |
| VEH (=1) | 0.085*** | | 0.302* |  |  |  |
|  | (0.018) | | (0.150) |  |  |  |
| VEHW (=1) | 0.085*** | | 0.101 |  |  |  |
|  | (0.018) | | (0.177) |  |  |  |
| Age |  | |  |  |  |  |
| Age 11 (=1) | 0.064* | | 0.094 | -0.011 | -0.129 | 0.030 |
|  | (0.032) | | (0.082) | (0.103) | (0.087) | (0.105) |
| Age 12 (=1) | 0.018 | | 0.057 | -0.011 | -0.126 | -0.014 |
|  | (0.030) | | (0.074) | (0.097) | (0.079) | (0.095) |
| Age 13 (=1) | -0.001 | | 0.010 | 0.023 | -0.087 | 0.030 |
|  | (0.029) | | (0.073) | (0.092) | (0.077) | (0.094) |
| Age 14 (=1) | -0.022 | | 0.015 | 0.010 | -0.125 | -0.028 |
|  | (0.028) | | (0.071) | (0.092) | (0.077) | (0.091) |
| Age 15 (=1) | Ref | | ref |  |  | - |
|  |  | |  |  |  |  |
| Grade attainment (completed grades) | 0.006 | | 0.013 | -0.013 | -0.017 | 0.002 |
|  | (0.006) | | (0.016) | (0.020) | (0.019) | (0.020) |
| Cognitive test score | 0.004† | | 0.001 | -0.000 | 0.008 | 0.002 |
|  | (0.002) | | (0.005) | (0.006) | (0.007) | (0.006) |
| Mother completed primary school (=1) | -0.012 | | -0.042 | 0.062 | 0.027 | 0.022 |
|  | (0.012) | | (0.033) | (0.040) | (0.038) | (0.040) |
| Father completed primary school (=1) | 0.003 | | 0.072† | -0.106** | -0.098** | -0.062 |
|  | (0.014) | | (0.042) | (0.047) | (0.047) | (0.050) |
| Lives with both parents (=1) | 0.019† | | 0.001 | 0.027 | 0.036 | 0.014 |
|  | (0.012) | | (0.030) | (0.036) | (0.036) | (0.037) |
| Household wealth quintile | 0.000 | | 0.003 | 0.005 | -0.012 | -0.006 |
|  | (0.004) | | (0.011) | (0.013) | (0.013) | (0.013) |
| Constant | 0.764*** | |  |  |  | 0.683** |
|  | (0.056) | |  |  |  | (0.134) |
|  |  | |  |  |  |  |
| N | 2390 | | 2390 |  |  |  |
| P-value overall F-test | <0.001 | | <0.001 |  |  |  |
| P-value for F-test on treatment arms | 0.001 | | 0.151 |  |  |  |
| P-value for F-test on all interactions with study arms |  | | 0.510 |  |  |  |
| P-value for F-test on interactions with study arm |  | |  | 0.613 | 0.456 | 0.920 |

Notes: Column (1) presents OLS coefficients for the linear probability model of a binary variable (=1) for resurvey at the two-year follow-up, estimated with robust standard errors. Column (2) is the LPM controlling for the same set of variables (2a) as well as interactions between each right-side variable and a binary 0/1 variable for study arms 2, 3 and 4. *** p<0.001, ** p<0.01, * p<0.05, † p<0.1

**Appendix Table 4b. Wajir correlates of two-year follow-up survey response, by study arm**

| Dependent variable: Interviewed at two-year follow-up (=1) | | (1) | (2a) | (2b) | (2c) | (2d) |
| --- | --- | --- | --- | --- | --- | --- |
|  |  | | X | Study arm 2*X | Study arm 3*X | Study arm 4*X |
| Study arms: V-only (ref) |  | |  |  |  |  |
| VE (=1) | -0.011 | | 0.102 |  |  |  |
|  | (0.034) | | (0.121) |  |  |  |
| VEH (=1) | 0.034† | | 0.026 |  |  |  |
|  | (0.020) | | (0.110) |  |  |  |
| VEHW (=1) | 0.041* | | 0.180* |  |  |  |
|  | (0.020) | | (0.077) |  |  |  |
| Age |  | |  |  |  |  |
| Age 10 (=1) | 0.089*** | | 0.130* | -0.076 | 0.050 | -0.089 |
|  | (0.027) | | (0.049) | (0.080) | (0.075) | (0.067) |
| Age 11 (=1) | 0.043* | | 0.045 | -0.035 | 0.075 | -0.017 |
|  | (0.022) | | (0.043) | (0.060) | (0.068) | (0.056) |
| Age 12 (=1) | 0.014 | | 0.009 | -0.053 | 0.113 | -0.006 |
|  | (0.023) | | (0.051) | (0.068) | (0.071) | (0.064) |
| Age 13 (=1) | 0.010 | | 0.033 | -0.069 | 0.070 | -0.065 |
|  | (0.022) | | (0.044) | (0.062) | (0.064) | (0.058) |
| Age 14 (=1) | ref | | ref |  |  |  |
|  |  | |  |  |  |  |
| Enrolled in current school year (=1) | 0.149*** | | 0.170** | -0.009 | 0.015 | -0.070 |
|  | (0.025) | | (0.060) | (0.082) | (0.080) | (0.069) |
| Grade attainment (completed grades) | 0.014*** | | 0.027** | -0.017 | -0.021** | -0.016* |
|  | (0.004) | | (0.006) | (0.011) | (0.007) | (0.009) |
| Cognitive test score | 0.001 | | -0.004 | -0.002 | 0.009 | 0.012** |
|  | (0.002) | | (0.005) | (0.007) | (0.006) | (0.006) |
| Father attended any school (=1) | -0.007 | | -0.023 | 0.037 | 0.017 | -0.008 |
|  | (0.028) | | (0.049) | (0.090) | (0.069) | (0.078) |
| Lives with both parents (=1) | 0.004 | | 0.001 | 0.040 | -0.033 | -0.012 |
|  | (0.015) | | (0.033) | (0.050) | (0.041) | (0.041) |
| Household wealth quintile | -0.002 | | 0.007 | -0.010 | -0.013 | -0.005 |
|  | (0.007) | | (0.010) | (0.022) | (0.012) | (0.013) |
| District: South (ref) |  | |  |  |  |  |
| East (=1) | 0.039* | | 0.047 | 0.031 | 0.008 | -0.083* |
|  | (0.018) | | (0.031) | (0.051) | (0.050) | (0.041) |
| West (=1) | 0.018 | | 0.070* | -0.076 | -0.030 | -0.074† |
|  | (0.037) | | (0.029) | (0.139) | (0.055) | (0.039) |
| Constant | 0.674*** | |  |  |  | 0.601** |
|  | (0.044) | |  |  |  | (0.068) |
|  |  | |  |  |  |  |
| N | 2147 | | 2147 |  |  |  |
| P-value overall F-test | <0.001 | | <0.001 |  |  |  |
| P-value for F-test on treatment arms | 0.147 | | 0.072 |  |  |  |
| P-value for F-test on all interactions with study arms |  | | 0.002 |  |  |  |
| P-value for F-test on interactions with study arm |  | |  | 0.681 | 0.048 | 0.007 |

Notes: See notes to Appendix Table 4a. *** p<0.001, ** p<0.01, * p<0.05, † p<0.1

**Appendix Table 5: Kibera estimated intent-to-treat effects, additional results for individual outcomes**

|  | | | (1) | | (2) | (3) | (4) | (5) |
| --- | --- | --- | --- | --- | --- | --- | --- | --- |
|  | | V-Only two-year follow-up  Mean | | | VE  Estimate | VEH  Estimate | VEHW  Estimate | VE-VEH-VEHW Pooled  Estimate |
| Violence Prevention | | |  | |  |  |  |  |
| Experienced violence by a male in the past year (=1) | | | 0.422 | | -0.088** | -0.059* | -0.042 | -0.063* |
| 95% CI | | |  | | [-0.14, -0.03] | [-0.12, 0.00] | [-0.10, 0.02] | [-0.11, -0.01] |
| P-value | | |  | | 0.003 | 0.042 | 0.165 | 0.010 |
| False discovery rate adjusted q-value | | |  | | 0.024 | 0.179 | 0.256 | 0.044 |
| Extended controls regression estimate | | |  | | -0.089** | -0.060* | -0.044 | -0.064** |
| Weighted regression estimate | | |  | | -0.086** | -0.058* | -0.040 | -0.061* |
| Gender equitable attitudes z-score^1^ | | | 0.000 | | -0.036 | 0.054 | 0.078 | 0.032 |
| 95% CI | | |  | | [-0.15, 0.08] | [-0.07, 0.17] | [-0.04, 0.19] | [-0.07, 0.13] |
| P-value | | |  | | 0.545 | 0.375 | 0.194 | 0.527 |
| False discovery rate adjusted q-value | | | |  | 0.843 | 0.581 | 0.261 | 0.561 |
| Extended controls regression estimate | | |  | | -0.050 | 0.053 | 0.076 | 0.026 |
| Weighted regression estimate | | |  | | -0.035 | 0.058 | 0.073 | 0.032 |
| Positive gender schooling attitudes z-score | | | 0.211 | | -0.024 | -0.091† | -0.137* | -0.084* |
| 95% CI | | |  | | [-0.12, 0.07] | [-0.19, 0.01] | [-0.24, -0.03] | [-0.16, 0.00] |
| P-value | | |  | | 0.631 | 0.076 | 0.010 | 0.042 |
| False discovery rate adjusted q-value | |  | | | 0.843 | 0.246 | 0.033 | 0.089 |
| Extended controls regression estimate | | |  | | -0.037 | -0.092† | -0.136** | -0.088* |
| Weighted regression estimate | | |  | | -0.018 | -0.096† | -0.142** | -0.086* |
| Education | | |  | |  |  |  |  |
| Grade attainment | | | 7.501 | | 0.052† | 0.048† | 0.067* | 0.056* |
| 95% CI | | |  | | [0.00, 0.11] | [-0.01, 0.10] | [0.01, 0.12] | [0.01, 0.10] |
| P-value | | |  | | 0.064 | 0.087 | 0.012 | 0.018 |
| False discovery rate adjusted q-value | |  | | | 0.364 | 0.246 | 0.034 | 0.051 |
| Extended controls regression estimate | | |  | | 0.050† | 0.054* | 0.066* | 0.057* |
| Weighted regression estimate | | |  | | 0.054† | 0.049† | 0.071** | 0.058* |
| Primary school complete (=1) | | | 0.517 | | 0.020 | 0.017 | 0.032 | 0.023 |
| 95% CI | | |  | | [-0.03, 0.07] | [-0.03, 0.07] | [-0.02, 0.08] | [-0.02, 0.06] |
| P-value | | |  | | 0.430 | 0.519 | 0.214 | 0.284 |
| False discovery rate adjusted q-value | | |  | | 0.843 | 0.596 | 0.261 | 0.323 |
| Extended controls regression estimate | | |  | | 0.002 | 0.012 | 0.025 | 0.013 |
| Weighted regression estimate | | |  | | 0.024 | 0.014 | 0.032 | 0.023 |
| Enrolled in current school year (=1) | 0.959 | | | | 0.005 | 0.011 | 0.020† | 0.012 |
| 95% CI | | |  | | [-0.02, 0.03] | [-0.01, 0.03] | [0.00, 0.04] | [-0.01, 0.03] |
| P-value | | |  | | 0.644 | 0.309 | 0.050 | 0.194 |
| False discovery rate adjusted q-value | |  | | | 0.843 | 0.527 | 0.094 | 0.244 |
| Extended controls regression estimate | | |  | | 0.004 | 0.011 | 0.019† | 0.011 |
| Weighted regression estimate | | |  | | 0.005 | 0.012 | 0.021* | 0.013 |
| Conditional primary school complete (=1)^2^ [n=1,104] | | | 0.887 | | 0.037 | 0.020 | 0.070** | 0.042† |
| 95% CI | | |  | | [-0.01, 0.09] | [-0.03, 0.07] | [0.02, 0.12] | [0.00, 0.09] |
| P-value | | |  | | 0.155 | 0.440 | 0.003 | 0.056 |
| False discovery rate adjusted q-value | | |  | | 0.660 | 0.596 | 0.013 | 0.100 |
| Extended controls regression estimate | | |  | | 0.025 | 0.012 | 0.056* | 0.031 |
| Weighted regression estimate | | |  | | 0.037 | 0.021 | 0.071** | 0.043† |
| Conditional transition to secondary school (=1)^3^ [n=1,131] | | | 0.836 | | 0.036 | 0.035 | 0.071* | 0.047† |
| 95% CI | | |  | | [-0.02, 0.10] | [-0.02, 0.09] | [0.01, 0.13] | [0.00, 0.10] |
| P-value | | |  | | 0.245 | 0.254 | 0.014 | 0.067 |
| False discovery rate adjusted q-value | |  | | | 0.696 | 0.527 | 0.034 | 0.105 |
| Extended controls regression estimate | | |  | | 0.026 | 0.029 | 0.059* | 0.038 |
| Weighted regression estimate | | |  | | 0.036 | 0.036 | 0.071* | 0.048† |
| Health | | |  | |  |  |  |  |
| Knows the most fertile period during menstrual cycle (=1) | | | 0.087 | | 0.016 | 0.019 | 0.022 | 0.019 |
| 95% CI | | |  | | [-0.02, 0.05] | [-0.02, 0.05] | [-0.01, 0.06] | [-0.01, 0.05] |
| P-value | | |  | | 0.380 | 0.302 | 0.230 | 0.201 |
| False discovery rate adjusted q-value | | |  | | 0.843 | 0.527 | 0.261 | 0.244 |
| Extended controls regression estimate | | |  | | 0.016 | 0.019 | 0.023 | 0.019 |
| Weighted regression estimate | | |  | | 0.015 | 0.019 | 0.022 | 0.019 |
| Knows method of modern contraception^1^ (=1) [n=2,175] | | | 0.555 | | -0.007 | 0.130*** | 0.119*** | 0.081** |
| 95% CI | | |  | | [-0.06, 0.05] | [0.07, 0.19] | [0.06, 0.18] | [0.03, 0.13] |
| P-value | | |  | | 0.824 | 0.000 | 0.000 | 0.001 |
| False discovery rate adjusted q-value | | |  | | 0.876 | 0.001 | 0.001 | 0.013 |
| Extended controls regression estimate | | |  | | -0.009 | 0.130*** | 0.120*** | 0.080** |
| Weighted regression estimate | | |  | | -0.007 | 0.133*** | 0.114*** | 0.081** |
| SRH knowledge z-score^1^ [n=1,948] | | | 0.000 | | 0.016 | 0.213** | 0.158* | 0.130* |
| 95% CI | | |  | | [-0.11, 0.14] | [0.09, 0.34] | [0.03, 0.29] | [0.02, 0.24] |
| P-value | | |  | | 0.800 | 0.001 | 0.016 | 0.015 |
| False discovery rate adjusted q-value | | |  | | 0.876 | 0.007 | 0.034 | 0.051 |
| Extended controls regression estimate | | |  | | 0.006 | 0.210** | 0.160* | 0.126* |
| Weighted regression estimate | | |  | | 0.014 | 0.218** | 0.155* | 0.131* |
| General self-efficacy z-score | | | 0.325 | | 0.152** | 0.030 | 0.055 | 0.079† |
| 95% CI | | |  | | [0.06, 0.25] | [-0.07, 0.13] | [-0.05, 0.16] | [-0.08, 0.12] |
| P-value | | |  | | 0.002 | 0.554 | 0.284 | 0.058 |
| False discovery rate adjusted q-value | |  | | | 0.024 | 0.596 | 0.302 | 0.100 |
| Extended controls regression estimate | | |  | | 0.149** | 0.030 | 0.057 | 0.079† |
| Weighted regression estimate | | |  | | 0.157** | 0.037 | 0.058 | 0.083* |
| Condom use self-efficacy z-score^1^ [n=1,777] | | | 0.000 | | 0.038 | 0.178** | 0.124† | 0.114* |
| 95% CI | | |  | | [-0.10, 0.17] | [0.05, 0.30] | [-0.01, 0.25] | [0.01, 0.22] |
| P-value | | |  | | 0.575 | 0.006 | 0.060 | 0.040 |
| False discovery rate adjusted q-value | |  | | | 0.843 | 0.035 | 0.103 | 0.041 |
| Extended controls regression estimate | | |  | | 0.035 | 0.174** | 0.123† | 0.111* |
| Weighted regression estimate | | |  | | 0.045 | 0.188** | 0.117† | 0.118* |
| Wealth creation | | |  | |  |  |  |  |
| Financial literacy z-score | | | -0.085 | | 0.014 | 0.045 | 0.381*** | 0.145** |
| 95% CI | | |  | | [-0.11, 0.13] | [-0.07, 0.16] | [0.26, 0.50] | [0.05, 0.24] |
| P-value | | |  | | 0.815 | 0.460 | 0.000 | 0.004 |
| False discovery rate adjusted q-value | |  | | | 0.876 | 0.596 | 0.001 | 0.023 |
| Extended controls regression estimate | | |  | | -0.007 | 0.040 | 0.379*** | 0.136** |
| Weighted regression estimate | | |  | | 0.009 | 0.037 | 0.369*** | 0.138** |
| Saved money in the past six months (=1) | | | 0.448 | | 0.000 | 0.017 | 0.202*** | 0.072** |
| 95% CI | | |  | | [-0.06, 0.06] | [-0.04, 0.08] | [0.14, 0.26] | [0.02, 0.12] |
| P-value | | |  | | 1.000 | 0.560 | 0.000 | 0.003 |
| False discovery rate adjusted q-value | | |  | | 1.000 | 0.596 | 0.001 | 0.023 |
| Extended controls regression estimate | | |  | | -0.004 | 0.017 | 0.202*** | 0.071** |
| Weighted regression estimate | | |  | | 0.005 | 0.018 | 0.205*** | 0.075** |
| Household-level outcomes | | |  | |  |  |  |  |
| Household wealth quintile [n=2,236] | | | 2.823 | | 0.101 | 0.123 | 0.105 | 0.110 |
| 95% CI | | |  | | [-0.06, 0.26] | [-0.03, 0.28] | [-0.06, 0.27] | [-0.02, 0.24] |
| P-value | | |  | | 0.214 | 0.125 | 0.201 | 0.102 |
| False discovery rate adjusted q-value | |  | | | 0.696 | 0.304 | 0.261 | 0.145 |
| Extended controls regression estimate | | |  | | 0.093 | 0.130 | 0.099 | 0.107 |
| Weighted regression estimate | | |  | | 0.112 | 0.129 | 0.111 | 0.117† |
| Expected girl to complete secondary school (=1) [n=2,193] | | | 0.994 | | 0.002 | -0.001 | 0.004 | 0.001 |
| 95% CI | | |  | | [-0.01, 0.01] | [-0.01, 0.01] | [0.00, 0.01] | [-0.01, 0.01] |
| P-value | | |  | | 0.629 | 0.777 | 0.304 | 0.682 |
| False discovery rate adjusted q-value | | | |  | 0.843 | 0.778 | 0.305 | 0.682 |
| Extended controls regression estimate | | |  | | 0.002 | -0.001 | 0.004 | 0.002 |
| Weighted regression estimate | | |  | | 0.002 | -0.002 | 0.004 | 0.001 |

Notes: The table reproduces the main OLS results shown in Table 4 (see notes for Table 4) in the text and also reports the associated P-values. Binary outcomes indicated by (=1) are linear probability models. Additional results reported include the false discovery rate adjusted q-value based on consideration of all 17 outcomes at once. Main regressions in the text Table 4 were estimated with robust standard errors and included controls for age and the outcome measured at baseline unless otherwise noted. The extended controls regression results reported here additionally control for baseline cognitive test score, school enrollment, grades attained, mother and father completion of primary school, whether the girl lives with both parents, wealth quintile and a binary variable indicating if any of these additional controls were imputed. The weighted regression results (without extended controls) reweight observations based on estimated probability of having been re-interviewed in the two-year follow-up (see note on attrition weight construction). N = 2,190; sample is smaller for some individual outcomes due to missing data as indicated. *** p<0.001, ** p<0.01, * p<0.05, † p<0.1

^1^ No baseline control for outcome variable available.

^2^ Among girls who had completed grade 6 but not yet grade 8 at baseline.

^3^ Among girls who had completed grade 6 but were not yet enrolled in secondary school at baseline.

**Appendix Table 6: Kibera estimated intent-to-treat effects, additional results for summary measures**

|  | (1) | | (2) | (3) | (4) | (5) |
| --- | --- | --- | --- | --- | --- | --- |
|  | V-Only two-year follow-up  Mean | | VE  Estimate | VEH  Estimate | VEHW  Estimate | VE-VEH-VEHW Pooled  Estimate |
| Violence Prevention |  | |  |  |  |  |
| *Violence prevention outcomes summary index z-score* | 0.000 | | 0.066 | 0.037 | -0.004 | 0.033 |
| 95% CI |  | | [-0.05, 0.19] | [-0.08, 0.16] | [-0.13, 0.12] | [-0.07, 0.13] |
| P-value |  | | 0.275 | 0.551 | 0.954 | 0.510 |
| False discovery rate adjusted q-value | |  | 0.368 | 0.552 | 0.955 | 0.511 |
| Extended controls regression estimate |  | | 0.050 | 0.036 | -0.004 | 0.028 |
| Weighted regression estimate |  | | 0.068 | 0.034 | -0.011 | 0.030 |
| Education |  | |  |  |  |  |
| *Education outcomes summary index z-score (grade, primary, enroll)* | 0.000 | | 0.062 | 0.082 | 0.123* | 0.089* |
| 95% CI |  | | [-0.04, 0.17] | [-0.02, 0.18] | [0.03, 0.22] | [0.00, 0.17] |
| P-value |  | | 0.248 | 0.107 | 0.013 | 0.042 |
| False discovery rate adjusted q-value |  | | 0.368 | 0.214 | 0.018 | 0.056 |
| Extended controls regression estimate |  | | 0.043 | 0.082† | 0.116* | 0.080† |
| Weighted regression estimate |  | | 0.068 | 0.088† | 0.129* | 0.095* |
| Health |  | |  |  |  |  |
| *Health outcomes summary index z-score* | 0.000 | | 0.113† | 0.306*** | 0.279*** | 0.233*** |
| 95% CI |  | | [0.00, 0.23] | [0.19, 0.42] | [0.16, 0.40] | [0.14, 0.33] |
| P-value |  | | 0.056 | 0.000 | 0.000 | 0.000 |
| False discovery rate adjusted q-value | |  | 0.226 | 0.001 | 0.001 | 0.001 |
| Extended controls regression estimate |  | | 0.102† | 0.302*** | 0.279*** | 0.228*** |
| Weighted regression estimate |  | | 0.114† | 0.317*** | 0.273*** | 0.236*** |
| Wealth creation |  | |  |  |  |  |
| *Wealth creation outcomes summary index z-score* | 0.000 | | 0.015 | 0.056 | 0.517*** | 0.194*** |
| 95% CI |  | | [-0.10, 0.13] | [-0.06, 0.17] | [0.40, 0.63] | [0.10, 0.29] |
| P-value |  | | 0.807 | 0.347 | 0.000 | 0.000 |
| False discovery rate adjusted q-value | |  | 0.808 | 0.463 | 0.001 | 0.001 |
| Extended controls regression estimate |  | | -0.005 | 0.052 | 0.516*** | 0.186*** |
| Weighted regression estimate |  | | 0.015 | 0.050 | 0.511*** | 0.191*** |

Notes: The table reproduces the main OLS results shown in Table 4 for summary outcomes, reporting additional information as in Appendix Table 5. See notes for Table 4 and Appendix Table 5. *** p<0.001, ** p<0.01, * p<0.05, † p<0.1.

**Appendix Table 7: Kibera estimated intent-to-treat effects for binary outcomes using**

**logistic regression**

|  | (1) | | (2) | (3) | (4) |
| --- | --- | --- | --- | --- | --- |
|  | V-Only two-year follow-up  Mean | | VE  Odds Ratio | VEH  Odds Ratio | VEHW  Odds Ratio |
| Violence Prevention |  | |  |  |  |
| Experienced violence by a male in the past year (=1) | 0.422 | | 0.680** | 0.774* | 0.837 |
| 95% CI |  | | [0.53, 0.88] | [0.60, 0.99] | [0.65, 1.08] |
| Education |  | |  |  |  |
| Primary school complete (=1) | 0.517 | | 1.131 | 1.096 | 1.203 |
| 95% CI |  | | [0.84, 1.51] | [0.82, 1.46] | [0.90, 1.60] |
| Enrolled in current school year (=1) | 0.959 | | 1.160 | 1.345 | 2.228† |
| 95% CI |  | | [0.58, 2.30] | [0.69, 2.62] | [0.98, 5.04] |
| Conditional primary school complete (=1)^2^ [n=1,104] | 0.887 | | 1.544 | 1.252 | 2.839** |
| 95% CI |  | | [0.85, 2.80] | [0.72, 2.17] | [1.41, 5.72] |
| Conditional transition to secondary school (=1)^3^ [n=1,131] | 0.836 | | 1.334 | 1.322 | 1.915* |
| 95% CI |  | | [0.83, 2.15] | [0.83, 2.11] | [1.14, 3.22] |
| Health |  | |  |  |  |
| Knows most fertile period during menstrual cycle (=1) | 0.087 | | 1.204 | 1.240 | 1.286 |
| 95% CI |  | | [0.79, 1.82] | [0.82, 1.87] | [0.85, 1.94] |
| Knows method of modern contraception^1^ (=1) [n=2,175] | 0.555 | | 0.973 | 1.824*** | 1.722*** |
| 95% CI |  | | [0.76, 1.25] | [1.41, 2.36] | [1.33, 2.23] |
| Wealth creation |  | |  |  |  |
| Saved money in the past six months (=1) | | 0.448 | 1.001 | 1.076 | 2.363*** |
| 95% CI |  | | [0.78, 1.28] | [0.84, 1.37] | [1.83, 3.05] |
| Household-level outcomes |  | |  |  |  |
| Expected girl to complete secondary school (=1) [n=2,193] | 0.994 | | 1.559 | 0.808 | 3.166 |
| 95% CI |  | | [0.26, 9.52] | [0.18, 3.60] | [0.32, 31.13] |
|  |  | |  |  |  |

Notes: The table reports two-year follow-up means for V-only in column 1, and the estimated ITT odds ratio effect for each study arm relative to V-only in columns 2–4 controlling for age and the outcome measured at baseline unless otherwise noted. Numbers in square brackets indicate 95% confidence intervals. Logistic regressions (in contrast to OLS presented in Table 4) were estimated with robust standard errors. N = 2,190; sample is smaller for some individual outcomes due to missing data as indicated. *** p<0.001, ** p<0.01, * p<0.05, † p<0.1

^1^ No baseline control for outcome variable available.

^2^ Among girls who had completed grade6 but had not yet completed grade 8 at baseline.

^3^ Among girls who had completed grade 6 but had not yet enrolled in secondary school at baseline.

**Appendix Table 8: Wajir estimated intent-to-treat effects, additional results for individual outcomes**

|  | | (1) | | | | (2) | (3) | (4) | (5) |
| --- | --- | --- | --- | --- | --- | --- | --- | --- | --- |
|  | | V-Only two-year follow-up  Mean | | | | VE  Estimate | VEH  Estimate | VEHW  Estimate | VE-VEH-VEHW Pooled  Estimate |
| Violence Prevention | |  | | | |  |  |  |  |
| Experienced violence by a male in the past year (=1) [n=1,878] | | 0.038 | | | | -0.006 | 0.015 | -0.022 | -0.004 |
| 95% CI | |  | | | | [-0.04, 0.03] | [-0.02, 0.05] | [-0.05, 0.01] | [-0.03, 0.03] |
| P-value | |  | | | | 0.774 | 0.422 | 0.112 | 0.787 |
| False discovery rate adjusted q-value | | |  | | | 0.775 | 0.537 | 0.241 | 0.848 |
| Extended controls regression estimate | | |  | | | -0.003 | 0.016 | -0.022 | -0.003 |
| Weighted regression estimate | |  | | | | -0.006 | 0.028 | -0.020 | 0.000 |
| Gender equitable attitudes z-score^1^ [n=1,903] | | 0.000 | | | | -0.171 | -0.208* | -0.018 | -0.132 |
| 95% CI | |  | | | | [-0.39, 0.05] | [-0.39, -0.02] | [-0.26, 0.22] | [-0.31, 0.05] |
| P-value | |  | | | | 0.127 | 0.029 | 0.882 | 0.146 |
| False discovery rate adjusted q-value | | |  | | | 0.179 | 0.068 | 0.882 | 0.211 |
| Extended controls regression estimate | | |  | | | -0.167 | -0.208* | -0.013 | -0.130 |
| Weighted regression estimate | |  | | | | -0.134 | -0.116 | 0.057 | -0.068 |
| Positive gender schooling attitudes z-score [n=1,878] | | 0.160 | | | | 0.156* | 0.099 | 0.068 | 0.107† |
| 95% CI | |  | | | | [0.00, 0.31] | [-0.05, 0.25] | [-0.08, 0.21] | [-0.02, 0.23] |
| P-value | |  | | | | 0.044 | 0.198 | 0.346 | 0.085 |
| False discovery rate adjusted q-value | | |  | | | 0.104 | 0.294 | 0.441 | 0.150 |
| Extended controls regression estimate | | |  | | | 0.196** | 0.118 | 0.104 | 0.138* |
| Weighted regression estimate | |  | | | | 0.182* | 0.096 | 0.075 | 0.120* |
| Education | |  | | | |  |  |  |  |
| Grade attainment | | 4.492 | | | | 0.259** | 0.194* | 0.181* | 0.211** |
| 95% CI | |  | | | | [0.10, 0.42] | [0.02, 0.36] | [0.01, 0.35] | [0.08, 0.34] |
| P-value | |  | | | | 0.002 | 0.026 | 0.036 | 0.002 |
| False discovery rate adjusted q-value | | | |  | | 0.012 | 0.068 | 0.168 | 0.011 |
| Extended controls regression estimate | | | |  | | 0.311*** | 0.200* | 0.198* | 0.235*** |
| Weighted regression estimate | |  | | | | 0.234** | 0.203* | 0.145† | 0.196** |
| Primary school complete (=1) | | 0.130 | | | | 0.010 | 0.021 | -0.029 | 0.001 |
| 95% CI | |  | | | | [-0.05, 0.07] | [-0.05, 0.09] | [-0.08, 0.03] | [-0.05, 0.05] |
| P-value | |  | | | | 0.756 | 0.545 | 0.307 | 0.984 |
| False discovery rate adjusted q-value | | |  | | | 0.775 | 0.636 | 0.430 | 0.984 |
| Extended controls regression estimate | | |  | | | 0.021 | 0.040* | 0.004 | 0.022 |
| Weighted regression estimate | |  | | | | 0.025 | 0.006 | -0.032 | 0.001 |
| Enrolled in current school year (=1) | 0.808 | | | | | 0.144*** | 0.070* | 0.084** | 0.099*** |
| 95% CI | |  | | | | [0.09, 0.20] | [0.01, 0.13] | [0.02, 0.14] | [0.05, 0.15] |
| P-value | |  | | | | 0.000 | 0.025 | 0.006 | 0.000 |
| False discovery rate adjusted q-value | | | |  | | 0.001 | 0.068 | 0.042 | 0.001 |
| Extended controls regression estimate | | |  | | | 0.146*** | 0.072* | 0.085** | 0.100*** |
| Weighted regression estimate | |  | | | | 0.146*** | 0.086* | 0.087** | 0.108*** |
| Health | |  | | | |  |  |  |  |
| Knows the most fertile period during menstrual cycle (=1) [n=1,878] | | 0.049 | | | | -0.031† | -0.021 | -0.013 | -0.021 |
| 95% CI | |  | | | | [-0.06, 0.00] | [-0.05, 0.01] | [-0.04, 0.02] | [-0.05, 0.01] |
| P-value | |  | | | | 0.072 | 0.182 | 0.417 | 0.150 |
| False discovery rate adjusted q-value | | |  | | | 0.144 | 0.294 | 0.486 | 0.211 |
| Extended controls regression estimate | | | |  | | -0.029† | -0.019 | -0.010 | -0.019 |
| Weighted regression estimate | |  | | | | -0.028† | -0.015 | -0.010 | -0.018 |
| Knows method of modern contraception^1^ (=1) [n=1,848] | | 0.390 | | | | -0.097† | -0.103* | -0.096† | -0.099* |
| 95% CI | |  | | | | [-0.21, 0.02] | [-0.19, -0.01] | [-0.20, 0.01] | [-0.19, -0.01] |
| P-value | |  | | | | 0.090 | 0.027 | 0.079 | 0.027 |
| False discovery rate adjusted q-value | | |  | | | 0.159 | 0.068 | 0.238 | 0.062 |
| Extended controls regression estimate | | |  | | | -0.080 | -0.091* | -0.072 | -0.081* |
| Weighted regression estimate | |  | | | | -0.074 | -0.095* | -0.075 | -0.081* |
| SRH knowledge z-score^1,2^ [n=1,400] | | 0.000 | | | | 0.344* | 0.433** | 0.234† | 0.335** |
| 95% CI | |  | | | | [0.08, 0.60] | [0.17, 0.70] | [-0.03, 0.50] | [0.12, 0.55] |
| P-value | |  | | | | 0.010 | 0.002 | 0.085 | 0.003 |
| False discovery rate adjusted q-value | | | |  | | 0.041 | 0.026 | 0.238 | 0.012 |
| Extended controls regression estimate | | |  | | | 0.341* | 0.435** | 0.230† | 0.335** |
| Weighted regression estimate | |  | | | | 0.339* | 0.357* | 0.206 | 0.299** |
| General self-efficacy z-score [n=1,878] | | 0.990 | | | | -0.054 | -0.014 | -0.030 | -0.032 |
| 95% CI | |  | | | | [-0.25, 0.14] | [-0.22, 0.19] | [-0.22, 0.16] | [-0.19, 0.13] |
| P-value | |  | | | | 0.583 | 0.893 | 0.756 | 0.687 |
| False discovery rate adjusted q-value | | |  | | | 0.680 | 0.894 | 0.814 | 0.801 |
| Extended controls regression estimate | | |  | | | -0.022 | 0.003 | -0.004 | -0.008 |
| Weighted regression estimate | |  | | | | -0.053 | 0.019 | -0.035 | -0.024 |
| Wealth creation | |  | | | |  |  |  |  |
| Financial literacy z-score [n=1,878] | | 0.439 | | | | -0.069 | 0.030 | 0.185 | 0.050 |
| 95% CI | |  | | | | [-0.31, 0.17] | [-0.19, 0.25] | [-0.06, 0.43] | [-0.16, 0.26] |
| P-value | |  | | | | 0.564 | 0.793 | 0.137 | 0.639 |
| False discovery rate adjusted q-value | | |  | | | 0.680 | 0.855 | 0.241 | 0.801 |
| Extended controls regression estimate | | | | |  | -0.001 | 0.049 | 0.244* | 0.098 |
| Weighted regression estimate | |  | | | | -0.055 | 0.026 | 0.192 | 0.049 |
| Saved money in the past six months (=1) [n=1,878] | | 0.011 | | | | 0.030 | 0.047* | 0.409*** | 0.164*** |
| 95% CI | |  | | | | [-0.01, 0.07] | [0.01, 0.09] | [0.31, 0.51] | [0.09, 0.23] |
| P-value | |  | | | | 0.123 | 0.022 | 0.000 | 0.000 |
| False discovery rate adjusted q-value | | | |  | | 0.179 | 0.068 | 0.001 | 0.001 |
| Extended controls regression estimate | | | |  | | 0.042* | 0.049* | 0.418*** | 0.170*** |
| Weighted regression estimate | |  | | | | 0.036† | 0.045* | 0.423*** | 0.161*** |
| Household-level outcomes | |  | | | |  |  |  |  |
| Household wealth quintile [n=2,011] | | 3.226 | | | | -0.434* | -0.247 | -0.211 | -0.296† |
| 95% CI | |  | | | | [-0.83, -0.04] | [-0.64, 0.14] | [-0.54, 0.12] | [-0.59, 0.00] |
| P-value | |  | | | | 0.032 | 0.210 | 0.210 | 0.050 |
| False discovery rate adjusted q-value | | |  | | | 0.090 | 0.294 | 0.327 | 0.101 |
| Extended controls regression estimate | | | |  | | -0.424* | -0.246 | -0.186 | -0.283† |
| Weighted regression estimate | |  | | | | -0.389* | -0.143 | -0.143 | -0.234 |
| Expected girl to complete secondary school (=1) [n=2,007] | | 0.861 | | | | 0.058* | 0.050† | 0.043 | 0.050* |
| 95% CI | |  | | | | [0.01, 0.10] | [0.00, 0.10] | [-0.01, 0.10] | [0.01, 0.09] |
| P-value | |  | | | | 0.012 | 0.070 | 0.134 | 0.022 |
| False discovery rate adjusted q-value | | |  | | | 0.041 | 0.140 | 0.241 | 0.062 |
| Extended controls regression estimate | | | |  | | 0.074** | 0.052* | 0.056* | 0.060** |
| Weighted regression estimate | |  | | | | 0.055** | 0.048† | 0.032 | 0.046* |

Notes: The table reproduces the main OLS results shown in Table 5 (see notes for Table 5) in the text and also reports the associated P-values. Binary outcomes indicated by (=1) are linear probability models. Additional results reported include the false discovery rate adjusted q-value based on consideration of all 14 outcomes at once. Main regressions in the text Table 5 were estimated with standard errors clustered at the village level and included controls for district per the stratified randomization, age and the outcome measured at baseline unless otherwise noted. The extended controls regression results reported here additionally control baseline cognitive test score, school enrollment, grades attained, whether father had attended school, whether the girl lives with both parents, wealth quintile and a binary variable indicating if any of these additional controls were imputed. The weighted regression results (without extended controls) reweight observations based on estimated probability of having been re-interviewed in the two-year follow-up (see note on attrition weight construction). N = 1,909; sample is smaller for some individual outcomes due to missing data as indicated. *** p<0.001, ** p<0.01, * p<0.05, † p<0.1.

^1^ No baseline control for outcome variable available.

^2^ Non-response for one or more items on the scale ranged from 24–30% across study arms and was higher for younger girls.

**Appendix Table 9: Wajir estimated intent-to-treat effects, additional results for summary measures**

|  | (1) | | (2) | (3) | (4) | (5) |
| --- | --- | --- | --- | --- | --- | --- |
|  | V-Only two-year follow-up  Mean | | VE  Estimate | VEH  Estimate | VEHW  Estimate | VE-VEH-VEHW Pooled  Estimate |
| Violence Prevention |  | |  |  |  |  |
| *Violence prevention outcomes summary index z-score* [n=1,878] | 0.000 | | 0.030 | -0.101 | 0.104 | 0.010 |
| 95% CI |  | | [-0.15, 0.21] | [-0.29, 0.08] | [-0.07, 0.27] | [-0.15, 0.17] |
| P-value |  | | 0.731 | 0.283 | 0.229 | 0.896 |
| False discovery rate adjusted q-value |  | | 0.795 | 0.378 | 0.306 | 0.917 |
| Extended controls regression estimate |  | | 0.063 | -0.086 | 0.136 | 0.035 |
| Weighted regression estimate |  | | 0.069 | -0.094 | 0.143† | 0.040 |
| Education |  | |  |  |  |  |
| *Education outcomes summary index z-score* | 0.000 | | 0.302*** | 0.189* | 0.122 | 0.203** |
| 95% CI |  | | [0.16, 0.44] | [0.02, 0.36] | [-0.03, 0.27] | [0.08, 0.32] |
| P-value |  | | 0.000 | 0.031 | 0.109 | 0.001 |
| False discovery rate adjusted q-value |  | | 0.001 | 0.063 | 0.219 | 0.003 |
| Extended controls regression estimate |  | | 0.313*** | 0.198* | 0.120† | 0.208*** |
| Weighted regression estimate |  | | 0.329*** | 0.188* | 0.112† | 0.216*** |
| Health |  | |  |  |  |  |
| *Health outcomes summary index z-score* [n=1,878] | 0.000 | | -0.026 | 0.066 | -0.014 | 0.009 |
| 95% CI |  | | [-0.22, 0.17] | [-0.13, 0.26] | [-0.22, 0.19] | [-0.17, 0.19] |
| P-value |  | | 0.795 | 0.508 | 0.890 | 0.917 |
| False discovery rate adjusted q-value | |  | 0.795 | 0.508 | 0.891 | 0.917 |
| Extended controls regression estimate |  | | 0.019 | 0.091 | 0.031 | 0.048 |
| Weighted regression estimate |  | | 0.001 | 0.060 | -0.006 | 0.018 |
| Wealth creation |  | |  |  |  |  |
| *Wealth creation outcomes summary index z-score* [n=1,878] | 0.000 | | 0.167 | 0.349* | 2.852*** | 1.135*** |
| 95% CI |  | | [-0.14, 0.47] | [0.03, 0.66] | [2.14, 3.56] | [0.63, 1.64] |
| P-value |  | | 0.284 | 0.030 | 0.000 | 0.000 |
| False discovery rate adjusted q-value |  | | 0.568 | 0.063 | 0.001 | 0.001 |
| Extended controls regression estimate |  | | 0.285† | 0.373* | 2.951*** | 1.208*** |
| Weighted regression estimate |  | | 0.212 | 0.337* | 2.944*** | 1.119*** |

Notes: The table reproduces the main OLS results shown in Table 5 for summary outcomes, reporting additional information as in Appendix Table 7. See notes for Table 5 and Appendix Table 7. *** p<0.001, ** p<0.01, * p<0.05, † p<0.1.

*Note on attrition weight construction*

We calculated inverse probability weights (IPW) for each site (and for individual- and household-level survey variables separately) as follows. First, we constructed approximately 60 variables from each baseline survey (imputing the less than 1% of missing values with local area or village medians). In addition to variables in Appendix Tables 2 and 3, these included other individual level variables such as measures of residential mobility and a wide range of housing characteristics and ownership of household-level assets described in Austrian et al. (2015). In Wajir, we also included a binary variable for nine clusters in which there were security concerns during fieldwork. Second, we ran bivariate ordinary least squares (OLS) regressions on a binary variable of being interviewed at the two-year follow-up for each variable (separately for the V-only sample and for the VE, VEH and VEHW samples). We retained for potential inclusion in the weight construction all variables significant at P<0.1. Third, each variable, along with variables for age in years (and district in Wajir) was interacted with a binary variable of treatment in VE, VEH and VEHW (as in Appendix Table 4) so that the weights could be calculated separately for each study arm. Fourth, we estimated the probability of being interviewed on this set of baseline predictors. To account for collinearity between predictors, the baseline predictor set was further limited by conducting stepwise selection of variables with backward elimination and using the adjusted R^2^ as the information criteria. Variables for study arm and age (and district in Wajir) were fixed in the regressions. At each step, the iterative procedure removes from the model the predictor that most improves the information criterion until there is no variable whose removal improves it, implemented using the ‘vselect’ command in Stata (Lindsey and Sheather 2010). Fifth, using the resulting specification for each site and survey type, we predicted for each observation the probability of having been re-interviewed and constructed the IPW. Last, for Wajir we multiplied those weights with sample population weights based on the size of each cluster. In Kibera, sample weights for the individual analyses ranged from 0.88 to 1.67 (25^th^ percentile 1.02, 50^th^ 1.07, 75^th^ 1.15). In Wajir where they also incorporate population weights, weights ranged from 0.88 to 7.96 (25^th^ 1.21, 50^th^ 1.37, 75^th^ 1.73).

Austrian, K, Muthengi, E, Riley, T, Mumah, J, Kabiru C and Abuya B. (2015). AGI-K Baseline Report. Nairobi: Population Council.

Lindsey, C. and S. Sheather. (2010). “Variable selection in linear regression.” *The Stata Journal*, 10(4): 650–669.

**Appendix Table 10: Wajir estimated intent-to-treat effects for binary outcomes using**

**logistic regression**

|  | (1) | (2) | (3) | (4) |
| --- | --- | --- | --- | --- |
|  | V-Only two-year follow-up  Mean | VE  Odds Ratio | VEH  Odds Ratio | VEHW  Odds Ratio |
| Violence Prevention |  |  |  |  |
| Experienced violence by a male in the past year (=1) [n=1,878] | 0.038 | 0.851 | 1.418 | 0.414† |
| 95% CI |  | [0.28, 2.58] | [0.59, 3.40] | [0.17, 1.04] |
| Education |  |  |  |  |
| Primary school complete (=1) [n=1,908] | 0.130 | 1.087 | 1.244 | 0.700 |
| 95% CI |  | [0.57, 2.06] | [0.62, 2.50] | [0.36, 1.38] |
| Enrolled in current school year (=1) | 0.808 | 6.160*** | 2.269* | 3.234** |
| 95% CI |  | [3.26, 11.63] | [1.13, 4.55] | [1.63, 6.41] |
| Health |  |  |  |  |
| Knows most fertile period during menstrual cycle (=1) [n=1,878] | 0.049 | 0.353† | 0.558 | 0.744 |
| 95% CI |  | [0.10, 1.20] | [0.26, 1.20] | [0.36, 1.54] |
| Knows method of modern contraception^1^ (=1) [n=1,848] | 0.390 | 0.639† | 0.617* | 0.642† |
| 95% CI |  | [0.38, 1.07] | [0.41, 0.93] | [0.39, 1.05] |
| Wealth creation |  |  |  |  |
| Saved money in the past six months (=1) [n=1,878] | 0.011 | 3.781* | 5.679** | 68.905*** |
| 95% CI |  | [1.14, 12.56] | [1.82, 17.77] | [22.82, 208.0] |
| Household-level outcomes |  |  |  |  |
| Expected girl to complete secondary school (=1) [n=2,007] | 0.861 | 1.898** | 1.694† | 1.635 |
| 95% CI |  | [1.19, 3.04] | [0.91, 3.14] | [0.88, 3.04] |
|  |  |  |  |  |

Notes: The table reports two-year follow-up means for V-only in column 1, and the estimated ITT odds ratio effect for each study arm relative to V-only in columns 2–4 controlling for the 2009 district per the stratified randomization, age and the outcome measured at baseline unless otherwise noted. Numbers in square brackets indicate 95% confidence intervals. Logistic regressions (in contrast to OLS presented in Table 5) were estimated with standard errors clustered at the village level. N = 1,909; sample is smaller for some individual outcomes due to missing data as indicated. *** p<0.001, ** p<0.01, * p<0.05, † p<0.1

^1^ No baseline control for outcome variable available.

**Appendix Table 11: Two-year follow-up outcomes variable definitions**

| Variable | Survey items | Survey instrument |
| --- | --- | --- |
| **Violence prevention and gender attitudes domain** | |  |
| *Experienced violence by a male in the past year*  =1 if answered yes to any one of the 15 examples; 0 otherwise. | Has any male done any of the following things to you in the past year:   1. Say or do something to humiliate you in front of others 2. Threaten to hurt or harm you or someone close to you 3. Insult you or make you feel bad about yourself 4. Push you, shake you, or throw something at you 5. Slap you 6. Twist your arm or pull your hair 7. Punch you with his fist or something that could hurt you 8. Kick you, drag you, or beat you up 9. Try to choke you or burn you on purpose 10. Threatened to attack you with a knife or other weapon 11. Attacked you with a weapon 12. Touched you in a sexual way (e.g., kissing, grabbing, or fondling), when you did not want them to 13. Try to have sexual intercourse with you when you did not want to but did not succeed 14. Physically forced you to have sexual intercourse even when you did not want to 15. Forced you to perform sexual acts when you did not want to | Individual |
| *Gender equitable attitudes score*  Summative score ranging from 0–10 with one point for each answer corresponding to the more gender equitable response as shown. | Agree or disagree with the statements:   1. Girls should avoid playing sports with boys because they get hurt easily (disagree=1) 2. Boys should be raised tough so they can overcome any difficulty in life (disagree=1) 3. Girls should avoid raising their voice to be lady like (disagree=1) 4. Boys should always defend themselves even if it means fighting (disagree=1) 5. Girls are expected to be humble (disagree=1) 6. Girls should always fight back if boys try to take advantage of them (agree=1) 7. Girls need their parents’ protection more than boys (disagree=1) 8. Boys should be able to show their feelings without fear of being teased (agree=1) 9. Boys who behave like girls are considered weak (disagree=1) 10. It's important for boys to show they are tough (disagree=1) | Individual |
| *Gender equitable schooling attitudes*  Summative score ranging from 0–4 with one point for each answer corresponding to the more gender equitable response as shown. | Agree or disagree with the statements:   1. It is as important for girls to complete secondary school as it is for boys (agree=1) 2. When a family cannot afford to send all children to school, it is better to send boys than girls (disagree=1) 3. A 16-year-old girl should get married when she finds an appropriate partner, even if she is still in school (disagree=1) 4. Girls are as intelligent as boys (agree=1) | Individual |
| *Violence prevention outcomes summary index z-score* | Constructed using *experienced violence by a male in the past year* (Kibera only), *gender equitable attitudes score*, and *gender equitable schooling attitudes* |  |
|  |  |  |
| **Education domain** |  |  |
|  |  |  |
| *Grade attainment*  Number of completed grades, 0–12. | What was the highest level of school you attended: primary or secondary? What is the highest class you completed at primary school? What is the highest class you completed at secondary school? | Individual |
| *Primary school complete*  =1 if completed; 0 otherwise. | Has completed primary school (class 8) | Individual |
| *School enrollment*  =1 if enrolled; 0 otherwise. | Have you attended school at any time during [the 2017 school year]? | Individual |
| *Conditional primary school completion*  =1 if completed grade 8; 0 otherwise. Variable only defined for those who had completed grade 6 or 7 at baseline. | Respondents who had completed class 6 or 7 (but not yet class 8) at baseline had completed class 8 at follow-up | Individual |
| *Transition to secondary school*  =1 if enrolled in secondary; 0 otherwise. Variable only defined for those who had completed grade 6, 7 or 8 at baseline but had not yet enrolled in secondary. | Respondents who had completed class 6, 7 or 8 (but had not enrolled in secondary) at baseline had enrolled in secondary at follow-up | Individual |
|  |  |  |
| *Literate in Swahili and English*  =1 if read all four sentences correctly without interruption; 0 otherwise.  (Not an outcome variable but summarized in baseline tables.) | Read aloud the following sentences in Swahili:   1. Ukulima ni kazi ngumu. 2. Mtoto anasoma kitabu.   Sentences in English:   1. Parents love their children. 2. Farming is hard work. | Individual literacy assessment |
| *Education outcomes summary index z-score* | Constructed using *grade attainment*, *primary school complete* (Kibera only), and *school enrollment* |  |
|  |  |  |
| **Health domain** |  |  |
| *Knows most fertile period during menstrual cycle*  =1 if knows; 0 otherwise. | From one menstrual period to the next, are there certain days when a woman is more likely to become pregnant if she has sexual relations? Is this time just before her period begins, during her period, right after her period has ended, or two weeks after her period? (two weeks after her period=1) | Individual |
| *Knows method of modern contraception*  =1 if spontaneously named a modern contraceptive method; 0 otherwise. | I would like to talk about family planning – the various ways or methods that a couple can use to delay or avoid a pregnancy. Which ways or methods have you heard about? Pill, IUD, injectables, implants, male condom, female condom, emergency contraception | Individual |
| *SRH knowledge score*  Summative score ranging from 0–7 with one point for each correct statement about SRH correctly identified as a myth or a fact. | State if the following are facts or myths:   1. One cannot get pregnant with one sexual act (myth=1) 2. Contraceptives are for married women (myth=1) 3. Use two condoms for double protection (myth=1) 4. Menstrual blood means a woman is dirty (myth=1) 5. Contraceptive use is harmful for health (myth=1) 6. Contraceptive pills make women barren (myth=1) 7. Sexual feelings are normal (fact=1) | Individual |
| *General self-efficacy score*  Summative score ranging from 0–6 with one point for each statement agreed to. | I am going to read you some statements, please tell me if you agree or disagree with them:   1. I can always manage to solve difficult problems if I try hard enough. (agree=1) 2. I am confident that I could handle unexpected events very well. (agree=1) 3. Because of the help I can get, I know how to manage unexpected situations. (agree=1) 4. I can solve most problems if I make the necessary effort. (agree=1) 5. If I am in trouble, I can usually think of a solution. (agree=1) 6. I can usually handle any situation that comes my way. (agree=1) | Individual  Alpha Kibera: 0.59  Wajir: 0.73 |
| *Condom use self-efficacy score*  Summative score ranging from 5–25 based on responses to the questions scored 1–5 each. | I am going to read you some statements, please tell me if you are very unsure=1, somewhat unsure=2, somewhat sure=3, sure=4, very sure=5:   1. I could carry a condom with me in case I needed one 2. I could use a condom each time I and my partner had sex 3. I could talk about using a condom if I were unsure about my partner's feelings on condoms 4. I could talk a partner into using a condom when we have sexual intercourse 5. I could say no to sex if my partner refused to use a condom | Individual  Alpha  Kibera: 0.85 |
| *Health outcomes summary index z-score* | Constructed using *knows most fertile period during menstrual cycle*, *knows method of modern contraception*, *SRH knowledge score*, *general self-efficacy score*, and *condom use self-efficacy score* (Kibera only) |  |
|  |  |  |
| **Wealth creation domain** |  |  |
|  |  |  |
| *Financial literacy score*  Summative score ranging from 0–10 with one point for each correct or savings-oriented answer. | I’m going to read you a story and then ask you some questions about the story: "Each week, Anna sits down and plans what she will earn and spend in the next week. She writes down all the places where she will get money and all the things she will spend it on. Then she is able to see if she has enough money for all of what she wants to buy."   1. Do you have such a plan? 2. What would you call that kind of plan?   I’m going to read you another story and then ask you some questions about it: ''Imani is 17 and and lives with her mother and her younger sister. Her older sister Mary is married and lives in another town, three hours away. Mary just had a baby boy, and Imani is eager to visit her sister. Imani will need to save money for transport and a small gift for the baby. A cute little hat would be perfect! But she can’t take money from her savings because she is saving that money to start her own business. Imani’s dream is to start a small catering business. Hopefully, her neighbor will employ her to work extra days in her hotel so she can get the money she needs for her trip."   1. What is one of Imani's short term financial goals? 2. What is Imani’s long term financial goal? 3. What is one formal way of saving your money? 4. What is one informal way of saving your money? 5. Grace would like to buy a new notebook for the next school term which starts in eight weeks. If the notebook costs KSH 100 and she can save KSH10 each week, will she reach her goal? 6. In the situation I described in the previous question, if Grace figured out how much she needed to save each week, and for how many weeks she needed to save in order to reach her goal, what would that be called? 7. If Grace discovered that she couldn’t reach her goal with that plan, what changes could she make so she would still reach her goal? 8. Do you agree or disagree with the following statement: “Only people with a lot of money can save”? | Individual |
|  |  |  |
| *Saved money in the past six months*  =1 if yes; 0 otherwise. | In the past six months, have you saved, or put money aside to use at a later time? | Individual |
| *Wealth creation outcomes summary index z-score* | Constructed using *financial literacy score* and *saved money in the past six months* |  |
|  |  |  |
| **Household-level domain** |  |  |
| *Household expects girl to complete secondary school*  =1 if highest level is secondary school or higher, 0 if not | What is the highest level of school that you expect [NAME] to complete? | Household |
| *Household wealth quintile*  Quintile of the first component of the PCA with 1 representing the lowest and 5 the highest. | Principal components analysis (PCA) estimated using the following ten items:     1. Does this household own any livestock, herds, or other farm animals or poultry? 2. Does any member of this household own any agricultural land? 3. Does your household have a television? 4. Does your household have a clock or watch? 5. Does your household have a mosquito net? 6. How many rooms in this household are used for sleeping? 7. In the last one month, was there a day that the household went without food because there wasn't enough food in the household? 8. Does your household have enough savings or something(s) to sell if you need 1000 shillings? 9. Does your household have enough savings or something(s) to sell if you need 5000 shillings? 10. Does your household have enough savings or something(s) to sell if you need 10000 shillings? | Household |
|  |  |  |
